# Supplementary material for: A combined experimental and modelling approach for the Weimberg pathway optimisation
Source: Nat Commun. 2020 Feb 27;11:1098. doi: 10.1038/s41467-020-14830-y (PMC7046635; doi:10.1038/s41467-020-14830-y)
Supplement: Supplementary file 1 — Supplementary Information [file 41467_2020_14830_MOESM1_ESM.pdf]

A combined experimental and modelling approach for the Weimberg pathway optimisation

Shen *et al.*

Supplementary Note 1. Model construction and optimization

Initial rate kinetics

D-xylose dehydrogenase (XDH): For the NAD^+ dependent reduction of D-xylose (XYL) to D-xylonolactone (XLAC), an irreversible, random order reaction mechanism, with product inhibition (Eq. **1**) was used to describe XDH activity. The equation was fitted to initial rate kinetics for the purified enzyme (Supplementary Table 3, Supplementary Fig. 4). For estimating the binding constants of the products, initial rate experiments in the presence of added D-xylonolactone and NADH were performed (Supplementary Fig. 4c, d).

$$v\text{XDH} = \frac{V_{M\text{XDH}} \frac{\text{NAD}^+}{K_{\text{XDHNAD}^+}} \frac{\text{XYL}}{K_{\text{XDHXYL}}}}{\left(1 + \frac{\text{NAD}^+}{K_{\text{XDHNAD}^+}} + \frac{\text{NADH}}{K_{\text{XDHNADH}}}\right)\left(1 + \frac{\text{XYL}}{K_{\text{XDHXYL}}} + \frac{\text{XLAC}}{K_{\text{XDHXLAC}}}\right)} \qquad (\mathbf{1})$$

Xylonolactonase (XLA): D-xylonolactone (XLAC) produced by XDH can be metabolised enzymatically by the XLA, or in a non-enzymatic way. The kinetics for D-xylonolactone metabolism was followed in presence and absence of XLA, by linking D-xylonolactone dependent D-xylonate (XA) production to D-xylonate dehydratase (XAD), 2-keto-3-deoxy-D-xylonate dehydratase (KDXD) and α-ketoglutarate semialdehyde dehydrogenase (KGSADH). The non-enzymatic degradation of D-xylonolactone was linearly dependent on its substrate (Supplementary Fig. 5a) and the data were fitted to a linear equation (eq. **2**), assuming mass action kinetics, while the lactonase showed a hyperbolic saturation curve and the kinetics (Supplementary Fig. 5b) were fitted to a Michaelis-Menten mechanism with product inhibition (eq. **3**). Equations 2 and 3 could describe the kinetic data well with the estimated parameter values (Supplementary Table 4). Since the assay for initial rate kinetics is linked to D-xylonate, we could not measure product inhibition with this assay, and fitted the binding constant for D-xylonate in the conversion assays (Supplementary Fig. 9b).

$$v\text{XLANE} = k_{XLA} \cdot \text{XLAC} \qquad (\mathbf{2})$$

$$v\text{XLA} = \frac{V_{M\text{XLA}} \cdot \frac{\text{XLAC}}{K_{\text{XLAXLAC}}}}{1 + \frac{\text{XLAC}}{K_{\text{XLAXLAC}}} + \frac{\text{XA}}{K_{\text{XLAXA}}}} \qquad (\mathbf{3})$$

D-xylonate dehydratase (XAD): D-xylonate (XA) formed by XLA can be converted into 2-keto-3-deoxy-D-xylonate (KDX) by the D-xylonate dehydratase (XAD). We assumed Michaelis-Menten kinetics (eq. **4**) for the reaction and fitted the equation to the initial rate kinetics for the

enzyme (Supplementary Table 5), measured using an off-line colorimetric assay. The kinetic data and fitted rate equation are shown in Supplementary Fig. 6. The product inhibition constant for 2-keto-3-deoxy-D-xylonate and inhibition constant for NADH were from incubations made with the two effectors: 10 mM additional 2-keto-3-deoxy-D-xylonate resulted in 50% reduction of activity for 10 mM D-xylonate conversion, and 3 mM NADH resulted in a 28% reduction of activity.

$$v\mathrm{XAD} = \frac{V_{M\mathrm{XAD}} \cdot \frac{\mathrm{XA}}{K_{\mathrm{XADXA}}}}{\left(1 + \frac{\mathrm{XA}}{K_{\mathrm{XADXA}}} + \frac{\mathrm{KDX}}{K_{\mathrm{XADKDX}}}\right)\left(1 + \frac{\mathrm{NADH}}{K_{i\mathrm{XADNADH}}}\right)} \qquad (\mathbf{4})$$

2-keto-3-deoxy-D-xylonate dehydratase (KDXD): 2-keto-3-deoxy-D-xylonate (KDX) is converted to *α*-ketoglutarate semialdehyde (KGSA) by KDXD. The enzyme showed a hyperbolic saturation curve for its substrate but was found to be sensitive to a number of metabolites. In addition to the direct product α-ketoglutarate semialdehyde, the enzyme was inhibited by D-xylonate, *α*-ketoglutarate (KG), pyruvate and lactate, grouped as inh_i in eq. **5**. Product inhibition for the individual metabolites and combination of metabolites was determined in initial rate experiments in a linked KGSADH assay (Supplementary Fig. 7), while for α-ketoglutarate semialdehyde the binding constant was estimated in a conversion assay (Supplementary Fig. 10b). No inhibition was observed for D-xylose and D-xylonolactone (up to 5 mM concentrations).

$$v\mathrm{KDXD} = \frac{V_{M\mathrm{KDXD}} \cdot \frac{\mathrm{KDX}}{K_{\mathrm{KDXDKDX}}}}{\left(1 + \frac{\mathrm{KDX}}{K_{\mathrm{KDXDKDX}}} + \frac{\mathrm{KGSA}}{K_{\mathrm{KDXDKGSA}}}\right)\left(1 + \sum_{i=1}^{4} \frac{\mathrm{inh}_i}{K_{i\mathrm{KDXD}}}\right)} \qquad (\mathbf{5})$$

α-Ketoglutarate semialdehyde dehydrogenase (KGSADH): α-ketoglutarate semialdehyde (KGSA) formed by KDXD is oxidised by KGSADH to α-ketoglutarate (KG). We used a random order binding mechanism, for a 2 substrates 2 products, irreversible rate equation (eq. **6**) to describe the reaction kinetics. The rate equation was fitted to the experimental data set, including inhibition by the products NADH and KG, and 2-keto-3-deoxy-D-xylonate (see Supplementary Fig. 8). Note that the dissociation constant for NADH was roughly a factor 10 lower in the enzyme-KDX complex, i.e. 0.024, compared to 0.27 when no 2-keto-3-deoxy-D-xylonate was bound to the enzyme.

$$v\mathrm{KGSADH} = \frac{V_{M\mathrm{KGSADH}} \cdot \frac{\mathrm{NAD^+}}{K_{\mathrm{KGSADHNAD^+}}} \cdot \frac{\mathrm{KGSA}}{K_{\mathrm{KGSADHKGSA}}}}{\left(1 + \frac{\mathrm{NAD^+}}{K_{\mathrm{KGSADHNAD^+}}} + \frac{\mathrm{NADH}}{K_{\mathrm{KGSADHNADH^*}}}\right)\left(1 + \frac{\mathrm{KGSA}}{K_{\mathrm{KGSADHKGSA}}} + \frac{\mathrm{KG}}{K_{\mathrm{KGSADHKG}}} + \frac{\mathrm{KDX}}{K_{\mathrm{KGSADHKDX}}}\right)} \qquad (\mathbf{6})$$

In vitro reconstituted pathway

For model validation we used NMR analyses of conversion assays in incubations; first incubations with sequentially added single enzymes (progress curves), and then for mixtures of enzymes (one-pot cascade analyses). Enzyme concentrations were chosen such that a good resolution was possible with the NMR, i.e. 5 mM conversions in circa 90 min time scale.

Sequential enzyme cascade

Each of the individual enzymes of the D-xylose degradation pathway was added sequentially to follow the conversion of 5 mM D-xylose through the pathway. The enzyme kinetic rate equations constructed on the basis of the initial rate characteristics were tested in their ability to predict the conversions. We had to fit product inhibition constants for XLA and KDXD, which cannot be assayed in initial rate kinetics (due to the nature of the linked assays).

D-xylose dehydrogenase (XDH): For the individual enzyme reactions an incubation was started with initial concentrations of substrates D-xylose (5 mM), NAD^+ (12 mM) and XDH (2.5 µg ml^{-1}), without XLA. The model prediction for the XDH conversion of D-xylose to D-xylonolactone, in combination with the non-enzymatic conversion of D-xylonolactone to D-xylonate, was well predicted by the model, based on the initial rate kinetics (Supplementary Fig. 9a, solid line). Note that the activity of XDH is significantly inhibited by both its products, D-xylonolactone and NADH. Without product inhibition a much faster rate of conversion is predicted (Supplementary Fig. 9a, dotted line), and of the two products the inhibition by NADH was most prominent (Supplementary Fig. 9a, dashed line).

Xylonolactonase (XLA): After 90 min, at the end of the XDH incubation, XLA (0.8 µg ml^{-1}) was added and the conversion of D-xylonolactone to D-xylonate was followed (Supplementary Fig. 9b). Without product inhibition the model prediction for the conversion was much faster than the experimentally measured conversion (Supplementary Fig. 9b, dashed line). Therefore, we included a product inhibition by D-xylonate into the model (Eq. 3) and fitted the binding constant on the conversion data (Supplementary Table 4), this resulted in a good description of the experimental data (Supplementary Fig. 9b, solid line).

D-xylonate dehydratase (XAD): At the end of the XLA incubation, XAD (6.5 µg ml^{-1}) was added and the conversion of D-xylonate to 2-keto-3-deoxy-D-xylonate was followed (Supplementary Fig. 10a). Product inhibition and inhibition by NADH (formed in the XDH

reaction) were included and resulted in a significant lowering of the conversion rate (Supplementary Fig. 10a). However, the model prediction for the conversion was still faster than the experimentally observed conversion. The fairly straight conversion profile in the experimental data indicate that it is not the product inhibition that is underestimated (this inhibition would increase from 0 to strong inhibition not resulting in a straight conversion line), but could be due to a stronger than estimated NADH inhibition. We are unsure why the enzyme activity would be lower in the conversion assays compared to the initial rate kinetics. The conditions are fairly similar, except for the enzyme concentration and the incubation time which have higher values (2 fold, and 50 fold respectively) in the conversion assay.

2-keto-3-deoxy-D-xylonate dehydratase (KDXD): After the XAD reaction, KDXD was added (3.3 µg ml^{-1}) and the conversion of 2-keto-3-deoxy-D-xylonate to α-ketoglutarate semialdehyde was followed (Supplementary Fig. 10b). Without product inhibition the model predicted a higher conversion rate than was observed experimentally (Supplementary Fig. 10b, dotted line) and we fitted α-ketoglutarate semialdehyde inhibition for KDXD (Eq. 5, Table 6). This resulted in a good description of the experimental data (Supplementary Fig. 10b, solid line).

α-Ketoglutarate semialdehyde dehydrogenase (KGSADH): After the KDXD, KGSADH was added (6.6 µg ml^{-1}) and the conversion of α-ketoglutarate semialdehyde to α-ketoglutarate was followed (Supplementary Fig. 11). The kinetic rate equation based on the initial rate kinetics gave a good prediction of the experimental data (Supplementary Fig. 11 solid line). As was observed for all enzymes in the system, the KGSADH was strongly inhibited by its products. In absence of product inhibition, the conversion would have been much faster (indicated with dotted lines in Supplementary Fig. 11). Similar as observed for the XDH, NADH contributed strongest to the product inhibition (Supplementary Fig. 11, dashed line).

One-pot cascade

After the pathway reconstitution via the sequential enzyme additions, we also analysed the conversion of D-xylose in a complete pathway. Three different perturbations were made: first, a reference state analysis, to check the model prediction for the complete pathway system and make final model adaptations. Subsequently, we solved the pathway inhibition caused by NADH via inclusion of an NAD^+ recycling system. Lastly, the optimal ratio of enzymes was designed for fastest conversion of D-xylose to α-ketoglutarate under different conditions.

Reference state analysis: For the reference state analysis, the five enzymes of the D-xylose degradation pathway were added together in concentrations close to those used in the sequential analysis: 2.5 µg ml^{-1} XDH, 0.8 µg ml^{-1} XLA, 8.67 µg ml^{-1}l XAD, 0.5 µg ml^{-1} KDXD, 10 µg ml^{-1} KGSADH. The system was analysed using ^{13}C and ^{1}H-NMR to follow the metabolism of 4.5 mM D-xylose and its conversion into α-ketoglutarate via the pathway intermediates (Main text Fig. 3A). With the rate equations that were constructed on the basis of the isolated reaction kinetics we could make a fairly accurate prediction of the first part of the pathway, but one parameter had to be adapted to make a good description of the complete pathway (Main text Fig. 3A). In contrast to the incubations with sequential addition of the enzymes, the conversion in the complete pathway did not run to completion, and specifically the conversion via KDXD was slow (high concentrations of 2-keto-3-deoxy-D-xylonate remaining), and the high α-ketoglutarate semialdehyde to α-ketoglutarate ratio suggested strong inhibition of KGSADH.

Activity of KDXD appeared to be strongly inhibited in the complete pathway, which was evident from the much lower 2-keto-3-deoxy-D-xylonate concentrations in the model prediction as compared to the experimentally measured values. Therefore, we tested the sensitivity of KDXD for all the pathway intermediates in initial rate kinetic experiments and found that the enzyme is sensitive to several of the intermediates (Supplementary Fig. 7, and Supplementary Table 6). However, these inhibitory effects are not strong enough to explain the reduced enzyme activity in the pathway analysis. We had to lower the activity to 25% of the isolated enzyme activity, which resulted in a much better description of 2-keto-3-deoxy-D-xylonate.

Model validation - pathway with NAD^+ recycling: Model analyses suggested that the incomplete conversion of D-xylose to α-ketoglutarate in the complete pathway analyses was due to NADH inhibition, and this was tested by inclusion of an NAD^+ recycling reaction to the system (10 U lactate dehydrogenase and 15 mM pyruvate).

Model validation - pathway without XLA: To further validate the model we analysed the effect of XLA omission on the pathway in the reference state, to test whether the non-enzymatic conversion of D-xylonolactone to D-xylonate limits the overall pathway flux in the absence of NAD^+ recycling (see also below).

Model application for rational pathway design: Subsequently, we analysed the optimal protein ratios for the fastest conversion of D-xylose to α-ketoglutarate, with a given total protein amount of 22.5 µg ml^{-1} (Supplementary Table 8). This is the same amount of protein as used in the conversion and pathway analyses, where it roughly took 600 min for complete conversion of 5 mM D-xylose with NAD^+ recycling (Main text Fig. 3B). When we distribute the protein amount over the different enzymes in the pathway for fastest conversion (with NAD^+ recycling), it was possible to achieve full conversion in 100 min (Main text Fig. 3D), The biggest change in enzyme concentrations between the reference state and the optimized state are the relative concentrations of KDXD and KGSADH, where a shift to KDXD makes the conversion of 2-keto-3-deoxy-D-xylonate, which accumulates in the reference state, to α-ketoglutarate semialdehyde faster. In contrast, when we analyze the optimal enzyme distributions for the pathway in absence of NAD^+ recycling, KGSADH is by far the most abundant enzyme (Supplementary Table 8). Under these conditions only 64% conversion is obtained in 600 min.

To confirm the predictions from the rational design but also to demonstrate that our model is of broad applicability we performed the fastest conversion rate experiment in presence of XLA and NAD^+ recycling using two different enzyme preparations. In addition to fresh enzyme solutions (duplicate) we used the Weimberg enzymes that were stored for around one year either frozen or at 4°C depending on protein stability (Supplementary Table 9) in order to demonstrate the predictive power of the simulation. All stored enzymes were still active but a considerable decrease in specific activity was observed (residual specific activity: XDH 52%, XLA 121%, XAD 86%, KDXD 65%, KGSADH 78%). The maximal velocity of the stored enzymes was determined and included in the model. The results (Main text Fig. 3D) show that the model predictions corresponded quite well to the experimental data obtained either with activities of fresh enzyme preparations or with the altered activities of the stored enzymes. This demonstrates that the model can be easily adopted to stored enzymes with changed enzymatic activities, novel enzyme preparations or different assay conditions.

Cell-free extract analysis

To analyse the Weimberg pathway under *in vivo* conditions, *C. crescentus* was grown in presence of D-xylose and D-glucose (Supplementary Fig. 12) and cells were harvested in the exponential phase (OD_{600} around 1) by centrifugation (15 min, 8150 x g, 4 °C). Afterwards, the cell pellets were resuspended with HEPES/NaOH buffer (pH 7.3, RT) supplemented with 300 mM NaCl to a final OD of 20 and were disrupted by sonication on ice for 3 x 5 min (1 min interval in between). Cell debris was removed by centrifugation (45 min, 21130 x g, 4°C). The protein concentration in cell-free extract (CFE) was determined using the Bradford assay with

BSA (Merck) as standard. Dialysis of CFE was not performed due to the inactivation of KGSADH.

The specific activities of the Weimberg enzymes were determined in the CFE (Supplementary Table 10). While all other enzyme activities were detected with 6-20 µg ml^{-1} CFE protein, for the XAD 30 µg ml^{-1} CFE protein as well as addition of Mn^{2+} (0.15 mM $MnCl_2$) was required and a specific activity of 0.173 ± 0.016 U mg^{-1} CFE protein was determined. However, since we observed a significant inhibition of the XAD activity to about one third of activity at higher protein concentration of 0.4 mg ml^{-1}, which is the same CFE protein concentration as used in the D-xylose conversion experiment, this activity was used for modelling (Supplementary Table 10, 11).

The model was tested in its capability to predict the *in vivo* conversions by inserting the specific activities of the enzymes measured in the CFE into the model. To convert the specific activity of the enzymes in the CFE to amounts of purified enzymes in the model, a number of calculations had to be made (Supplementary Table 11). First, the specific activity U mg^{-1} total protein was converted into a V_M, by inserting the specific activity and the assay conditions into the model rate equation and then solve for the V_M. The protein concentration can then be calculated by dividing the V_M per total protein by the V_M per purified protein, to yield the pure protein per total protein. In the model this value is then multiplied by the total protein per ml incubation volume (0.4 mg ml^{-1}) to yield the pure protein per ml in the assay.

The metabolite detection by NMR for the CFE was not possible due to the complexity of the samples (data not shown). Therefore, we used enzyme assays (in off-line analyses after protein precipitation) to quantify the metabolite concentrations in CFE conversions (Supplementary Table 12). Thus, we were able to determine D-xylose, D-xylonolactone + D-xylonate (due to non-enzymatic conversion of D-xylonolactone to D-xylonate these two metabolites could not be separated in the off-line analysis), 2-keto-3-deoxy-D-xylonate, α-ketoglutarate and we followed the extent of NAD^+ recycling via NADH and pyruvate determinations.

Supplementary Note 2. Intermediate verification and model validation by NMR

Both ^{1}H and ^{13}C spectral measurements were optimized to give one data point each every five minutes, which was found to be the optimum balance between fast observation of the kinetic behavior of the reaction and the intensity of the signal obtained. All ^{1}H-NMR spectra were recorded in 90 : 10 H_2O : D_2O at 600 MHz at 37°C and all ^{13}C-NMR spectra were recorded in 90 : 10 H_2O : D_2O at 151 MHz at 37°C. Integral regions were chosen which could be integrated clearly and were not obstructed by signals corresponding to the buffer or the solvent used. Studies on reference samples (commercially acquired) and enzymatically produced samples were compared to the samples measured and the integral regions chosen accordingly. Where possible, several integral regions were employed in order to verify the compounds. The integral of the buffer signal was set at a normalized value (according to 100 mM). All integral values corresponding to substrates under investigation were taken relative to the integral value of the buffer signal.

For D-xylonolactone, for which no signals in ^{1}H-NMR could be observed which could be integrated clearly, the concentration was calculated to be the difference to the maximum product concentration of 5 mM, assuming that all other components were accounted for. The presence of D-xylonolactone was confirmed by ^{13}C-NMR.

In case of the 2-keto-3-deoxy-D-xylonate, only two out of the three species could be observed clearly, the signals for the remaining species were found to be impacted by the buffer signal. It was found that in the enzymatically produced reference sample, the ketohydrate variety, the lactol variety and the keto variety were produced in a 1:1:1 ratio, therefore the two species observable were assumed to present 67% of the total concentration of 2-keto-3-deoxy-D-xylonate.

For the α-ketoglutarate semialdehyde, four different species were observed in the NMR, which were found to be present in a ratio of 2 : 1 : trace : 4 of aldohydrate : ketohydrate : bishydrate : lactone. Only the ketohydrate species could be followed reliably, therefore the ratio as observed before was assumed to be constant and a correction factor calculated to account for the unobservable species.

The α-ketoglutarate was observed in the ^{1}H-NMR analysis as the keto-form. The pathway which employed NAD^{+} recycling was the only one which went to completion, therefore the ^{13}C- NMR showed only a single product at the end of the reaction.

NMR spectral regions

Xylonolactone: The samples with added original compound and with enzymatically produced material both contain Xylonolactone and D-xylonate (probably from hydrolysis of the lactone) (ratio ca. 1:1 in both cases, signals for D-xylonate are listed below.

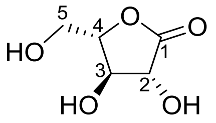

^{1}H NMR (600 MHz, D_2O, HEPES buffer, pH = 7.5) δ = 4.73 (dt, $^{3}J_{H4,H3}$ = 7.7 Hz, $^{3}J_{H4,H5}$ = 3.3 Hz, 1H, H-4), 4.70 (d, $^{3}J_{H2,H3}$ = 8.6 Hz, 1H, H-2), 4.62 (dd, $^{3}J_{H3,H2}$ = 8.6 Hz, $^{3}J_{H2,H4}$ = 7.7 Hz, 1H, H-3), 3.95 (d, $^{3}J_{H5,H4}$ = 3.3 Hz, 2zH, H-5).

^{13}C{^{1}H} NMR (151 MHz, D_2O, HEPES buffer, pH = 7.5) δ = 177.2 (C-1), 80.4 (C-4), 73.2 (C-3), 72.2 (C-2), 58.9 (C-5).

D-xylonate: The samples with added original compound and with enzymatically produced material both contain only D-xylonate.

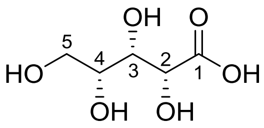

^{1}H NMR (600 MHz, D_2O, TRIS buffer, pH = 7.5) δ = 4.08 (d, $^{3}J_{H2,H3}$ = 2.6 Hz, 1H, H-2), 3.89 (dd, $^{3}J_{H3,H4}$ = 6.3 Hz, $^{3}J_{H3,H2}$ = 2.6 Hz, 1H, H-3), 3.82 (td, $^{3}J_{H4,H5'}$ = 6.4 Hz, $^{3}J_{H4,H3}$ = 6.4 Hz, $^{3}J_{H4,H5}$ = 3.8 Hz, 1H, H-4), 3.76 (dd, $^{2}J_{H5,H5'}$ = 11.9 Hz, $^{3}J_{H5,H4}$ = 3.8 Hz, 1H, H-5), 3.65 (dd, $^{2}J_{H5',H5}$ = 11.9 Hz, $^{3}J_{H5',H4}$ = 6.4 Hz, 1H, H-5´).

^{13}C{^{1}H} NMR (151 MHz, D_2O, TRIS buffer, pH = 7.5) δ = 178.6 (C-1), 72.81, 72.78 (C-2, C-4), 72.5 (C-3), 62.5 (C-5).

2-keto-3-deoxy-D-xylonate: No original compound was available. The sample with the enzymatically produced material contains 2-keto-3-deoxy-D-xylonate as a mixture of the ketone, the ketohydrate and the cyclic lactol (ratio ca. 1:1:1)[2]. The sample also contains NADH and NAD^{+}.

Keto-form:

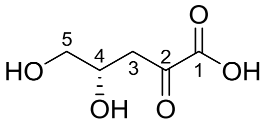

^{1}H NMR (600 MHz, D_2O, TRIS buffer, pH = 7.5) δ = 4.25-4.21 (m, 1H , H-4), 3.63 (dd, $^{3}J_{H5,H5'}$ = 11.8 Hz, $^{3}J_{H5,H4}$ = 4.1 Hz, 1H, H-5), 3.56 (dd, $^{3}J_{H5',H5}$ = 11.8 Hz, $^{3}J_{H5,H4}$ = 6.4 Hz, 1H, H-5´), 3.00 (dd, $^{3}J_{H3,H3'}$ = 17.1 Hz, $^{3}J_{H3,H4}$ = 4.3 Hz, 1H, H-3), 2.93 (dd, $^{3}J_{H3',H3}$ = 17.1 Hz, $^{3}J_{H3',H4}$ = 8.3 Hz, 1H, H-3).

^{13}C{^{1}H} NMR (151 MHz, D_2O, TRIS buffer, pH = 7.5) 203.8 (C-2), 67.6 (C-4), 65.1 (C-5), 42.9 (C-3) [signal for C-1 was not observed].

Ketohydrate:

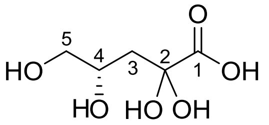

^{1}H NMR (600 MHz, D_2O, TRIS buffer, pH = 7.5) δ = 4.59 – 4.56 (m, 1H, H-4), 4.17 (dd, $^2J_{H5,H5´}$ = 9.6 Hz, $^3J_{H5,H4}$ = 4.2 Hz, 1H, H-5), 4.17 (d, $^2J_{H5´,H5}$ = 9.6 Hz, 1H, H-5´), 2.35 (dd, $^2J_{H3,H3´}$ = 14.4 Hz, $^3J_{H3,H4}$ = 5.7 Hz, 1H, H-3), 2.31 (dd, $^2J_{H3´,H3}$ = 14.4 Hz, $^3J_{H5,H4}$ = 2.5 Hz, 1H, H-3´).

^{13}C{^{1}H} NMR (151 MHz, D_2O, TRIS buffer, pH = 7.5) δ = 103.9 (C-2), 75.1 (C-5), 71.5 (C-4), 44.3 (C-3) [signal for C-1 was not observed].

Lactol:

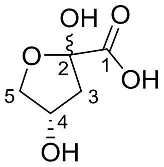

^{1}H NMR (600 MHz, D_2O, TRIS buffer, pH = 7.5) δ = 4.62-4.59 (m, 1H, H-4), 4.14 (dd, $^2J_{H5,H5}$ = 9.8 Hz, $^3J_{H5,H4}$ = 4.8 Hz, 1H, H-5), 4.03 (dd, $^2J_{H5´,H5}$ = 9.8 Hz, $^3J_{H5´,H4}$ = 1.8 Hz, 1H, H-5´), 2.49 (dd, $^2J_{H3,H3´}$ = 14.3 Hz, $^3J_{H3,H4}$ = 6.2 Hz, 1H, H-3), 2.49 (dd, $^2J_{H3,H3´}$ = 14.3 Hz, $^3J_{H3,H4}$ = 6.2 Hz, 1H, H-3), 2.08 (d, $^2J_{H3´,H3}$ = 14.3 Hz, 1H, H-3´).

^{13}C{^{1}H} NMR (151 MHz, D_2O, TRIS buffer, pH = 7.5) δ = 103.9 (C-2), 75.7 (C-5), 70.7 (C-4), 44.0 (C-3) [signal for C-1 was not observed].

α-ketoglutarate semialdehyde: The samples with added original compound and with enzymatically produced material both contain α-ketoglutarate semialdehyde as a mixture of the aldohydrate, the ketohydrate, the bishydrate and the lactone in varying amounts (ratio in case of added original compound ca. trace : 0.9 : 1 : trace, ratio in case of enzymatically produced material ca. 2 : 1 : trace : 4).[3]

Aldohydrate:

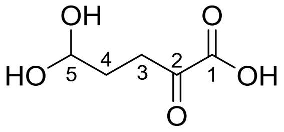

^{1}H NMR (600 MHz, D_2O, HEPES buffer, pH = 7.5) δ = 5.07 (s, 1H, H-5), 2.87 (s, 2H, H-3), 1.90 (s, 2H, H-4).

^{13}C{^{1}H} NMR (151 MHz, D_2O, HEPES buffer, pH = 7.5) δ = 90.1 (C-5), 36.9 (C-3), 30.8 (C-4) [signals for C-1 and C-2 not observed].

Ketohydrate:

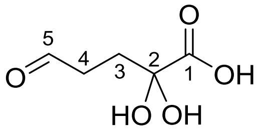

^{1}H NMR (600 MHz, D_2O, HEPES buffer, pH = 7.5) δ = 9.70 (s, 1H, H-5), 2.74 (s, 2H, H-4), 2.54 (bs, 2H, H-3).

^{13}C{^{1}H} NMR (151 MHz, D_2O, HEPES buffer, pH = 7.5) δ = 207.5 (b, C-5, from HSQC), 39.8 (C-4) [signals for C-1, C-2 and C-3 not observed].

Bishydrate:

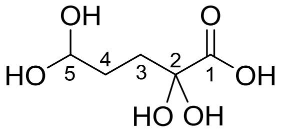

^{1}H NMR (600 MHz, D_2O, HEPES buffer, pH = 7.5) δ = 5.03 (s, 1H, H-5), 2.32 (bs, 2H, H-3), 1.85 (s, 2H, H-4).

^{13}C{^{1}H} NMR (151 MHz, D_2O, HEPES buffer, pH = 7.5) δ = 90.8 (C-5), 33.9 (C-4) [signals for C-1, C-2 and C-3 not observed].

Lactone:

It could not be clearly established whether the lactone is present in the 2-keto-form or as the 2-ketohydrate.

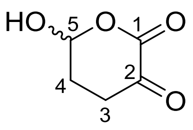

^{1}H NMR (600 MHz, D_2O, HEPES buffer, pH = 7.5) δ = 5.63 (s, 1H, H-5), 3.08, 2.29 (2 x bs, 2 x 1H, H-4, H-4´), 1.99 (bs, 2H, H-3, H-3´).

^{13}C{^{1}H} NMR (151 MHz, D_2O, HEPES buffer, pH = 7.5) δ = 99.4 (b, C-5, from HSQC), 32.4 (b, C-4), 31.9 (b, C-3) [signals for C-1 and C-2 not observed].

α-ketoglutarate: The samples with added original compound and with enzymatically produced material both contain α-ketoglutarate as a mixture of the keto-form and the lactone (ratio ca. 2:1 in both cases).[4]

Keto-form:

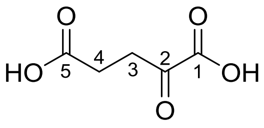

^{1}H NMR (600 MHz, D_2O, TRIS buffer, pH = 7.5) δ = 3.02 (t, $^3J_{H3,H4}$ = 6.9 Hz, 2H, H-3), 2.46 (t, $^3J_{H3,H4}$ = 6.9 Hz, 2H, H-3).

^{13}C{^{1}H} NMR (151 MHz, D_2O, TRIS buffer, pH = 7.5) δ = 205.3 (C-2), 181.2 (C-1/C-5, from HMBC), 35.9 (C-3), 30.70 (C-4).

Lactone:

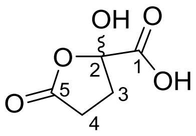

^{1}H NMR (600 MHz, D_2O, TRIS buffer, pH = 7.5) δ = 3.00 -2.97 (m, 1H, H-3/4), 2.46-2.41 (m, 3H, H-3/4).

^{13}C{^{1}H} NMR (151 MHz, D_2O, TRIS buffer, pH = 7.5) 34.3 (C-3, from HSQC), 30.65 (C-4) [signals for C-1, C-2 and C-5 not observed]

Integral regions for compound verification and calculation

Where possible, several integral regions were employed in order to verify the compounds. The integral of the buffer signal was set at a normalized value (according to 100 mM). All integral values corresponding to substrates under investigation were taken relative to the integral value of the buffer signal.

The approximate integral regions for all compounds are given in the Supplementary table 13. Depending on the NMR-spectra of each experiment, suitable integral regions were used. Due to signal overlaps, different line-widths and different baseline-quality, the exact integral regions have to be adapted for each experiment and not every species can be followed in each experiment.

In those cases, where only one hydration state (see above) is visible for a certain compound, a correction factor was applied based on the rations between the different hydration states as determined in the original NMR-experiments.

NMR instrument specification and pulse program code

The NMR measurements were performed on a Bruker AV3HD 600 MHz spectrometer with CyroProbe Prodigy and a BFFO sample head. ^{1}H NMR spectra were measured with an FID of 12,000 data points with an inverse-gated pulse of 16 scans and four dummies. Water signals were suppressed by an 8 ms pulse with excitation sculpting. The data was obtained over a range of 12 ppm. ^{1}H NMR spectra were manually phase-corrected and baseline-corrected before integration. ^{13}C spectra were measured in a 30° angle to obtain maximum signal-to-noise-ratio in the limited time available for kinetic investigation. The spectra were measured over 240 ppm over an FID of 53,000 data points (86 scans, 0 dummies). The pulse program was coded specifically for the experiments (Supplementary Data 1).

Supplementary Note 3. Production of KDG, KDX and KDGal in an industrial relevant scale

In order to demonstrate that the established enzyme cascade offers great biotechnological potential, we tested the production of 2-keto-3-deoxy sugar acids as well as (2S, 3R, 4S)-4-hydroxyisoleucine.

2-keto-3-deoxy-D-gluconic acid lithium salt

For the production of the 2-keto-3-deoxy-D-gluconic acid (KDG) lithium salt, a solution of 2.5 g (11.5 mmol) D-gluconic acid sodium salt in 125 ml H_2O deion. (pH 7.5) 16 units D-xylonat dehydratase from *Caulobacter crescentus* (CC-XAD, contains Mn^{2+}) were added and the mixture kept at 40°C while stirring. After 3 days the starting material has been consumed completely as shown by TLC (Silicagel 60, n-PrOH:NH_4OH:H_2O=6:1:1, $(NH_4)_2SO_4$, heating to 200°; r_f (gluconate) 0.07, r_f (KDG) 0.30). Concentration of the reaction mixture (rotary evaporator, 35°, vacuum) and then drying (r.t. high vacuum) gave 2.84 g of a resin which was flash chromatographed (silicagel 60, n-PrOH:NH_4OH:H_2O=6:1:1). The product containing fractions were pooled, concentrated and dried as above to give 2.2 g colorless foamy resin. This was solved in 5 ml H_2O deion, applied to a column containing 70 g Dowex 50WX8 in the lithium form and eluted with H_2O deion.. The eluate was freeze dried to give 2.35 g of KDG lithium salt as a slightly beige hygroscopic powder. TLC purity 97%; H_2O content (according to elemental analysis) 10-15% [w/w]; LC-MS-ESI: $[M-H]^-$ 177.0 Da.

2-keto-3-deoxy-D-xylonic acid lithium salt

For the production of 2-keto-3-deoxy-D-xylonic acid (KDX) lithium salt, a stirred solution of 2.1 g (14.2 mmol) D-xylono-1,4-lactone in 100 ml H_2O deion. was added with a pH titrator and at r.t. 1M NaOH to keep a pH of 7.5 (consumption ~14 ml, duration ~20 h). Then 33 units D-xylonat dehydratase from *Caulobacter crescentus* (CC-XAD, contains Mn^{2+}) were added and then the mixture was heated to 40°C and stirred for 25 h. TLC (Silicagel 60, n-PrOH:NH_4OH:H_2O=6:1:1, $(NH_4)_2SO_4$, heating to 200°) showed that ~2/3 of starting material (r_f ~0.13) had been converted and a single product (r_f ~0.35) was formed. Another 33 units Cbc-xylD were added and the mixture again stirred at 40°C for 24 h after which time the starting xylonate has been consumed completely according to TLC. The reaction mixture was concentrated (rotary evaporator, 35°, vacuum) and dried (r.t. high vacuum) to give 2.87 g of a foam. This was subjected to flash chromatography (silicagel 60, n-PrOH:NH_4OH:H_2O=6:1:1) and the product containing fractions were concentrated and dried as above to give 2.47 g of a foam which contained, according to ^1H-NMR, ~12% [w/w] acetic

acid. This was immediately solved in 20 ml H_2O deion., the solution treated with active carbon, filtered and applied to column containing 40g Dowex 50WX8 in the lithium form, eluted with H_2O deion. and eluate freeze dried to give 2.09 g of 2-keto-3-deoxy-D-xylonate as a foamy hygroscopic resin.

TLC purity 97%; $[\alpha]_D^{20}$ +3.4° (c=1 in H_2O); H_2O content (according to elemental analysis) ~13% [w/w]; LC-MS-ESI: $[M-H]^-$ 147.0 Da; ^1H- and ^{13}C-NMR (D_2O): shows three isoforms in full accordance to Archer and his coworkers[2].

2-keto-3-deoxy-D-galactonic acid lithium salt

For the production of 2-keto-3-deoxy-D-galactonic acid (KDGal) lithium salt, a stirred solution of 500 mg (2.55 mmol) D-galactonic acid lactone in 25 ml 0.5 M HEPES buffer at pH 7.5 14 units D-xylonat dehydratase from *Caulobacter crescentus* (CC-XAD, contains Mn^{2+}) were added and then the mixture was heated to 40°C. Every 5 h the pH was adjusted again to 7.5 with small amounts of 1 M NaOH (totally 2.2 ml). After 1 day the pH remained constant and after 2 days only traces of starting material could be detected on TLC (Silicagel 60, n-PrOH:NH_4OH:H_2O=6:1:1, $(NH_4)_2SO_4$, heating to 200°; r_f (starting material) ~0.08) r_f (product) ~0.30)). The mixture was concentrated (rotary evaporator, 35°, vacuum) and dried (r.t. high vacuum) to give 3 g of a viscous oil which was flash-chromatographed (silicagel 60, n-PrOH:NH_4OH:H_2O=6:1:1). The buffer containing fractions were again chromatographed to give in total 420 mg of a resin. This was solved in 4 ml H_2O deion., applied to a column containing 25 g Dowex 50WX8 in the lithium form, and eluted with 100 ml H_2O deion. The eluate was freeze dried to give 400 mg of KDGal lithium salt as a slightly yellow hygroscopic powder.

TLC purity: one spot; $[\alpha]_D^{20}$ +12.8° (c=1 in H_2O)[5]; LC-MS-ESI: $[M-H]^-$ 177.0 Da; ^1H- and ^{13}C-NMR (D_2O): shows predominantly three cyclic isoforms in a ratio of ~10:15:75 (dd, H_2-C(3)).

Supplementary Note 4. Production of (2S, 3R, 4S)-4-hydroxyisoleucine

(2S, 3R, 4S)-4-hydroxyisoleucine was produced at 30°C with all of the Weimberg pathway enzymes in combination with the L-isoleucine dioxygenase (BtDO) from *Bacillus thuringiensis*[6], using L-isoleucine and D-xylose as substrates and NAD^+ as cofactor. The reaction mixture (total volume 1 ml) contained 150 mM MES buffer (pH 7, 30°C), 0.6 U of each Weimberg pathway enzyme, 0.8 mg of BtDO, 8 mM NAD^+, 0.5 mM $FeSO_4$, 5 mM ascorbic acid, 2 mM L-isoleucine and 3 mM D-xylose. The mixture (without D-xylose) was pre-incubated at 30°C for 2 min and the reaction was started by the addition of D-xylose. 120 µl samples were taken at different time points (0, 30, 60, 90, 120, 180 min) and the reaction was immediately stopped by the addition of 12 µl 12% (v/v) trichloroacetic acid and transferred on ice for 10 min. Precipitated proteins were removed by centrifugation at 21130 x g, 4°C for 15 min. 100 µl of the supernatant was taken and neutralized by adding 4 µl of 2 M NaOH. The concentration of L-isoleucine and (2S, 3R, 4S)-4-hydroxyisoleucine in the neutralized samples were checked by HPLC using L-glutamate as standard[7].

Supplementary Table 1. Cloning and expression of the D-xylose degradation enzymes from *C. crescentus*[a].

Gene	Primer[b]	Vector	Overexpression strain	Expression
**CCNA_00862 (XAD)**	Fd (NdeI) 5'-CTAGATCATATGTCAACCGCACGCC<br>Rv (HindIII) 5'-GTACGAAGCTTTCAGTGGTTGTGGC	pET28b	Rosetta (DE3)	30°C overnight
**CCNA_00863 (XLA)**	Fd (NdeI) 5'- CCATAGCATATGACCGCTCAAGTCAC<br>Rv (BamHI) 5'-ATCTTGGATCCTTAGACAAGGCGGAC	pET15b	BL21 (DE3)-pRIL	21°C overnight
**CCNA_00864 (XDH)**	Fd (NdeI) 5'- GTCCTGCATATGTCCTCAGCCATCTATC<br>Rv (BamHI) 5'-CAATTGGATCCTCAACGCCAGCCG	pET15b	BL21 (DE3)-pRIL	37°C 4 h
**CCNA_00865 (KGSADH)**	Fd (NdeI) 5'- CAATCACATATGACCGACACCCTGC<br>Rv (BamHI) 5'- GTCTTGGATCCTTACGACCACGAGTAG	pET15b	Rosetta (DE3)	21°C overnight
**CCNA_00866 (KDXD)**	Fd (NdeI) 5'- GCATTGCATATGGGCGTGAGTGAATTC<br>Rv (BamHI) 5'- GAACTGGATCCTTAGAGGAGGCCGC	pET15b	BL21 (DE3)-pRIL	37°C 4 h

[a] pET system vectors were used for cloning and isopropyl-β-D-thiogalactopyranoside (IPTG) was used for induction.

[b] Fd: forward primer, Rv: reverse primer. The respective restriction enzyme is given in parentheses and the restriction sites are underlined.

Supplementary Table 2. Properties of the D-xylose degrading enzymes from *C. crescentus*[a].

Enzyme	D-xylose dehydrogenase	Xylonolactonase	D-xylonate dehydratase	2-keto-3-deoxy-D-xylonate dehydratase	α-ketoglutarate semialdehyde dehydrogenase
**UniProt ID**	B8H1Z0	A0A0H3C6P8	A0A0H3C6H6	A0A0H3C4T2	A0A0H3C801
**Locus tag**	CCNA_00864	CCNA_00863	CCNA_00862	CCNA_00866	CCNA_00865
**NCBI Gene ID**	7329904	7329903	7329902	7329906	7329905
**Subunit size (kDa)**	26.6	31.6	64.2	41.3	50.6
**Oligomerization**	Dimer	Monomer	Tetramer	Dimer	Tetramer
**V_{max} U mg^{-1}**	D-xylose (121.1)<br>L-arabinose (131.8)<br>D-ribose (6.2)	D-xylono-1,4-lactone (944.4)	D-xylonate (42.4)<br>D-gluconate (73.2)<br>D-galactonate (58.5)[b]	2-keto-3-deoxy-D-xylonate (107.1)	NAD^+ as cofactor:<br>KGSA (45.2)<br>SSA (0.3)<br>Glutaraldehyde (54.4)[b]<br>$NADP^+$ as cofactor:<br>KGSA (37.1)<br>SSA (0.4)<br>Glutaraldehyde (37.9)[b]
**K_m (mM)**	D-xylose (0.20)<br>NAD+ (0.17)<br>L-arabinose (71.09)	D-xylono-1,4-lactone (0.45)	D-xylonate (0.79)<br>D-gluconate (2.19)<br>D-galactonate (0.55, K_i = 58.14)[b]	2-keto-3-deoxy-D-xylonate (0.21)	NAD+ as cofactor:<br>KGSA (0.02)<br>SSA (0.40)<br>Glutaraldehyde (2.51, K_i = 6.44)[b]<br>KGSA as substrate:<br>NAD+ (0.59)<br>NADP+ (0.02)
**pH optimum (>50% activity)**	8.5 (6.0-9.5)	ND[c]	7.5 (7.0-8.5)	ND[c]	7.5 (6.0-9.0)
**Temperature optimum °C (>50% activity)**	55 (40-60)	ND[c]	55 (30-65)	ND[c]	40 (25-45)
**Thermostability**	ND[c]	ND[c]	75% residual activity (2 h at 55°C), 100% residual activity (2 h at 37°C)	ND[c]	100% residual activity (2.5 h at 37°C)

[a] Abbreviations: KGSA, α-ketoglutarate semialdehyde; SSA, succinic semialdehyde.

The V_{max} and K_m are calculated by Origin[1] using the classical Michaelis-Menten kinetics.

[b] For enzymes that show substrate inhibition, the following equation (**7**) was used for calculation:

$$v = \frac{V_{max} \cdot [S]}{K_m + [S]\left(1 + \frac{[S]}{K_i}\right)} \quad (\mathbf{7})$$

[c] ND: not determined.

Supplementary Table 3. Kinetic parameters of D-xylose dehydrogenase[a].

	Estimate	Standard error	Units
V_{MXDH}	$1.2 \cdot 10^{+2}$	2.9	(U mg^{-1})
K_{XDHXYL}	$2.0 \cdot 10^{-1}$	$1.7 \cdot 10^{-2}$	(mM)
K_{XDHNAD^+}	$1.6 \cdot 10^{-1}$	$1.6 \cdot 10^{-2}$	(mM)
$K_{XDHXLAC}$	$5.4 \cdot 10^{-1}$	$3.1 \cdot 10^{-2}$	(mM)
$K_{XDHNADH}$	$3.0 \cdot 10^{-2}$	$2.8 \cdot 10^{-3}$	(mM)

[a] For the estimation of the kinetic parameters of D-xylose dehydrogenase (XDH), Eq. 1 was fitted to initial rate kinetic data for XDH (Supplementary Fig. 4).

Supplementary Table 4. Kinetic parameters for xylonolactonase[a].

	Estimate	Standard error	Units
k_{XLA}	$7.2 \cdot 10^{-3}$	$7.7 \cdot 10^{-5}$	(min^{-1})
V_{MXLA}	$9.4 \cdot 10^{+2}$	$6.0 \cdot 10^{+1}$	(U mg^{-1})
$K_{XLAXLAC}$	$4.5 \cdot 10^{-1}$	$7.9 \cdot 10^{-2}$	(mM)
K_{XLAXA}	$3.7 \cdot 10^{-2}$	--	(mM)

[a] For the estimation of the kinetic parameters of D-xylonolactone (XLAC) conversion, Eqs. 2 and 3 were fitted to initial rate kinetic data for xylonolactonase (Supplementary Fig. 5). The binding constant for D-xylonate (XA) was fitted on the conversion assay data for xylonolactonase (XLA) (Supplementary Fig. 9b).

Supplementary Table 5. Kinetic parameters of D-xylonate dehydratase[a].

	Estimate	Standard error	Units
V_{MXAD}	$4.2 \cdot 10^{+1}$	$6.7 \cdot 10^{-1}$	(U mg^{-1})
K_{XADXA}	$7.9 \cdot 10^{-1}$	$4.9 \cdot 10^{-2}$	(mM)
K_{XADKDX}	$8.6 \cdot 10^{-1}$	--	(mM)
$K_{iXADNADH}$	$1.1 \cdot 10^{+1}$	--	(mM)

[a] For estimation of the kinetic parameters of D-xylonate dehydratase (XAD), Eq. 4 was fitted to initial rate kinetic data for XAD, measured using an off-line colorimetric assay (Supplementary Fig. 6).

Supplementary Table 6. Kinetic parameters of 2-keto-3-deoxy-D-xylonate dehydratase[a].

	Estimate	Standard error	Units
V_{MKDXD}	$1.1 \cdot 10^{+2}$	4.0	(U mg^{-1})
$K_{KDXDKDX}$	$2.1 \cdot 10^{-1}$	$3.5 \cdot 10^{-2}$	(mM)
$K_{KDXDKGSA}$	$2.9 \cdot 10^{-1}$	--	(mM)
$K_{iKDXDXA}$	$1.8 \cdot 10^{+1}$	1.2	(mM)
$K_{iKDXDKG}$	$1.5 \cdot 10^{+1}$	1.3	(mM)
$K_{iKDXDpyr}$	$1.8 \cdot 10^{+1}$	1.0	(mM)
$K_{iKDXDlac}$	$2.8 \cdot 10^{+1}$	1.1	(mM)

[a] For the estimation of the kinetic parameters, Eq. 5 was fitted to initial rate kinetic data for 2-keto-3-deoxy-D-xylonate dehydratase (KDXD) in a linked assay with α-ketoglutarate semialdehyde dehydrogenase (KGSADH) (Supplementary Fig. 7).

Supplementary Table 7. Kinetic parameters of α-ketoglutarate semialdehyde dehydrogenase[a].

	Estimate	Standard error	Units
$V_{M\mathrm{KGSADH}}$	$4.9\cdot10^{+1}$	1.9	(U mg^{-1})
$K_{\mathrm{KGSADHKGSA}}$	$2.2\cdot10^{-2}$	$3.1\cdot10^{-3}$	(mM)
K_{KGSADHKG}	$2.8\cdot10^{-1}$	$2.2\cdot10^{-2}$	(mM)
$K_{\mathrm{KGSADHKDX}}$	$2.1\cdot10^{-2}$	$9.6\cdot10^{-3}$	(mM)
$K_{\mathrm{KGSADHNAD^+}}$	$6.0\cdot10^{-1}$	$1.0\cdot10^{-1}$	(mM)
$K_{\mathrm{KGSADHNADH*}}$ (when complexed with KDX)	$2.7\cdot10^{-1}$ $2.4\cdot10^{-2}$	$7.0\cdot10^{-2}$ $1.1\cdot10^{-2}$	(mM)

[a] To estimate the kinetic parameters, Eq. 6 was fitted to initial rate kinetic data for α-ketoglutarate semialdehyde dehydrogenase (KGSADH) (Supplementary Fig. 8).

Supplementary Table 8. Predicted protein concentrations and ratios required for optimal conversion of D-xylose to α-ketoglutarate[a].

	Reference state (with NAD^+ recycling)	Fastest conversion (with NAD^+ recycling)	Fastest conversion (without NAD^+) [illegible]	Fastest conversion (with NAD^+ recycling, without XLA)
XDH (μg ml^{-1})	2.5	1.9	1.9	6.8
XLA (μg ml^{-1})	0.8	1.4	0.6	-
XAD (μg ml^{-1})	8.7	6.1	2.2	7.1
KDXD (μg ml^{-1})	0.5	9.4	3.3	6.1
KGSADH (μg ml^{-1})	10	3.7	14	2.5
Conversion rate (%)	100	100	99	98
Conversion time (min)	600	100	600	600

[a] The total enzyme amount is set to be 22.5 μg ml^{-1}.

Supplementary Table 9. Properties of the D-xylose degradation enzymes from *C. crescentus* before and after storage.

Enzyme	V_M (U mg^{-1})		Storage condition	Time of storage (month)	V_M (U mg^{-1}) (1st preparation after storage)	Residual activity (%)
	1st enzyme preparation	2nd enzyme preparation				
D-xylose dehydrogenase	128	124	4°C	16	66	52
Xylonolactonase	944	839	4°C	14	1147	121[a]
D-xylonate dehydratase	42	44	-70°C	14	38	86
2-keto-3-deoxy-D-xylonate dehydratase	107	79	4°C	11	70	65
α-ketoglutarate semialdehyde dehydrogenase	49	58	-70°C	14	38	78

[a] Due to the high specific activity of the xylonolactonase, the enzyme had to be significantly diluted in the enzyme assay, causing deviation in specific activity calculation.

Supplementary Table 10. Assay composition for enzyme activity determination in the cell-free extract of *C. crescentus*.

Object enzyme	CFE protein	Substrates	Metal ions	Auxiliary enzymes
**XDH**	8 µg ml^{-1}	D-xylose (5 mM)<br>NAD^+ (3 mM) /<br>$NADP^+$ (1 mM)	--	--
**XLA**	6 µg ml^{-1}	D-xylono-1,4-lactone (2mM)<br>NAD^+ (5 mM)	--	XAD (46.8 µg ml^{-1})<br>KDXD (28.4 µg ml^{-1})<br>KGSADH (31.0 µg ml^{-1})
**XAD**	400 µg ml^{-1}	D-xylonate (2 mM)<br>NAD^+ (5 mM)	--	KDXD (28.4 µg ml^{-1})<br>KGSADH (31.0 µg ml^{-1})
**XAD**	400 µg ml^{-1}	D-xylonate (2 mM)<br>NAD^+ (5 mM)	$MnCl_2$ (0.15 mM)	KDXD (28.4 µg ml^{-1})<br>KGSADH (31.0 µg ml^{-1})
**KDXD**	6 µg ml^{-1}	2-keto-3-deoxy-D-xylonate (3 mM)<br>[illegible]	--	KGSADH (31.0 µg ml^{-1})
**KGSADH**	20 µg ml^{-1}	KGSA (1 mM)<br>NAD^+ (5 mM) /<br>$NADP^+$ (1mM)	--	--

Supplementary Table 11. Calculation of protein concentrations in cell-free extract[a].

Enzyme	Specific activity (U mg^{-1} tot. prot.)	V_M (U mg tot. prot.$^{-1}$)	[Enzyme] (µg prot. mg tot. prot.$^{-1}$)
**XDH**	0.71	0.78	6.5
**XLA**	3.38	4.13	4.4
**XAD**	0.06	0.06	1.5
**KDXD**	0.81	0.98	9.2
**KGSADH**	0.55	0.63	13

[a] To estimate protein concentrations in CFE, the specific activities were converted to equivalent V_M values per total protein (tot. prot.) and then divided by the V_M of purified protein to yield the purified protein per total protein concentration. For the XAD the activity determined at 400 µg ml^{-1} cell-free extract (CFE) protein was used.

Supplementary Table 12. Assay composition for the enzymatic quantification of metabolite concentration[a].

	D-xylose	D-xylonate	2-keto-3-deoxy-D-xylonate	α-ketoglutarate semialdehyde	Pyruvate
**Cofactor**	2 mM NAD^+	2 mM NAD^+	2 mM NAD^+	2 mM NAD^+	0.5 mM NADH
**Enzymes**	0.7 U XDH	0.4 U XAD, 0.8 U KDXD, 0.5 U KGSADH	0.8 U KDXD, 0.5 U KGSADH	0.5 U KGSADH	1.0 U LDH
**Sample (protein removed)**	10 µl	10 µl	10 µl	10 µl	7µl
**Control 1**	No XDH in the mixture	No XAD in the mixture	No KDXD in the mixture	No KGSADH in the mixture	No LDH in the mixture
**Control 2**	Replace the 10 or 7 µl sample with 10 or 7 µl H_2O respectively in the reaction mixture				
**Buffer**	100 mM HEPES/NaOH (pH 7.5, 37°C)				
**H_2O**	Add up to total volume of 300 µl				

[a] The metabolite concentrations in the samples taken at different time points from the D-xylose conversion assay were determined photometrically (340 nm) after protein precipitation using the assays described. The assay mixtures were incubated at 37°C for 30 min and then 200 µl of each mixture was transferred into a microtiter plate (ROTH) to determine the absorbance (340 nm). The concentration of the metabolite was calculated according to the increase (for D-xylose, D-xylonate, 2-keto-3-deoxy-D-xylonate and α-ketoglutarate semialdehyde) or decrease (for pyruvate) of absorbance due to the production or consumption of NADH, respectively.

Supplementary Table 13. Integral regions available for quantification of the different compounds.

Molecular structure	Integral region I (assignment, number of observed protons)	Integral region II (assignment, number of observed protons)
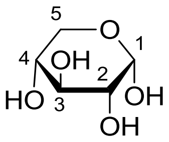<br>D-xylose (α-form)	5.31 – 5.28 ppm<br><br>(H-1, 1 H, 1/3 of the Xylose population, => 0.33 H)	3.57 – 3.51 ppm<br><br>H-4 (1 H, 1/3 of the Xylose population, => 0.33 H)
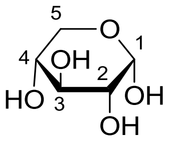<br>D-xylose (β-form)	3.46 – 3.39 ppm<br><br>H-3 (1 H, 2/3 of the Xylose population, => 0.33 H)	
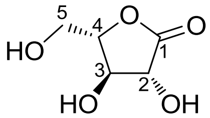<br>Xylonolactone	4.05 - 4.03 ppm<br><br>H-5 (2 H, only species)	
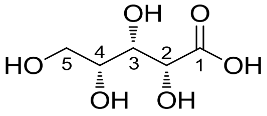<br>D-xylonate	3.88 – 3.84 ppm,<br><br>H-3 (1H, only species)	
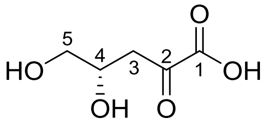<br>2-keto-3-deoxy-D-xylonate (KDX) (Ketone variety)	3.75 – 3.70 ppm<br><br>H-3 (1 H, 1/3 of the KDX population, => 0.33 H)	
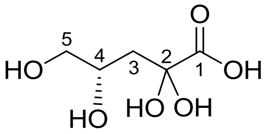<br>2-keto-3-deoxy-D-xylonate (KDX) (Ketohydrate variety)	2.46 – 2.38 ppm<br><br>H-3 (2 H, 1/3 of the KDX population, => 0.67 H)	
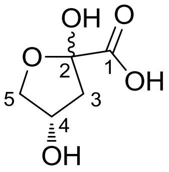<br>2-keto-3-deoxy-D-xylonate (Lactol variety)	2.20– 2.13 ppm<br><br>H-3´ (1 H, 1/3 of the KDX population, => 0.33 H)	
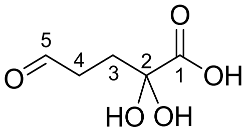<br>α-ketoglutarate semialdehyde (Ketohydrate variety)	9.83 – 9.80 ppm<br><br>H-5 (1 H, 1/7 of the KDX population, => 0.14 H)	
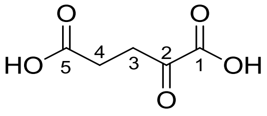<br>α-ketoglutarate (Keto-form)	2.62 – 2.47 ppm<br><br>H-3 (2 H, 2/3 of the KDX population, => 0.75 H)	

NAD+	9.53 – 9.40 ppm<br><br>(1 H, only one species)	
Pyruvate	2.53 – 2.44 ppm<br><br>(3 H, only one species)	
Lactate	1.58 – 1.34 ppm<br><br>(3 H, only one species)	
HEPES-buffer	4.07 – 3.93 ppm<br><br>(2 H, only one species)	

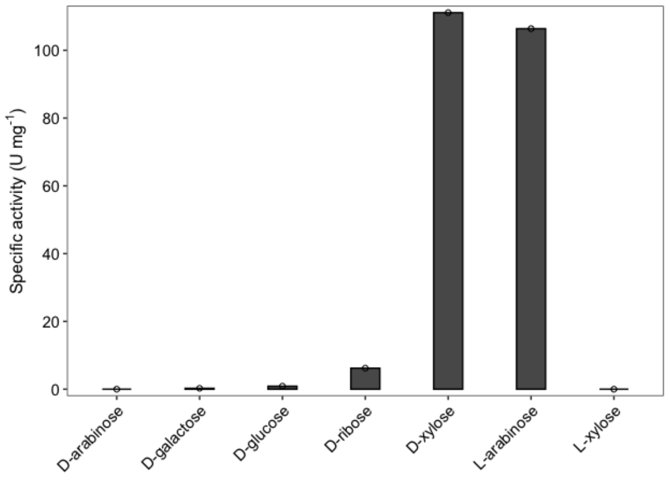

Supplementary Figure 1. Substrate spectrum of the D-xylose dehydrogenase. The specific activity was determined in 100 mM HEPES/NaOH (pH 7.5, 37°C) at 37°C using 30 mM of each sugar, 5 mM NAD^+ and 0.136-4 µg D-xylose dehydrogenase (XDH). $NADP^+$ is not utilized as cofactor by XDH. All measurements were performed without replicate (n=1). Source data are provided as a Source Data file.

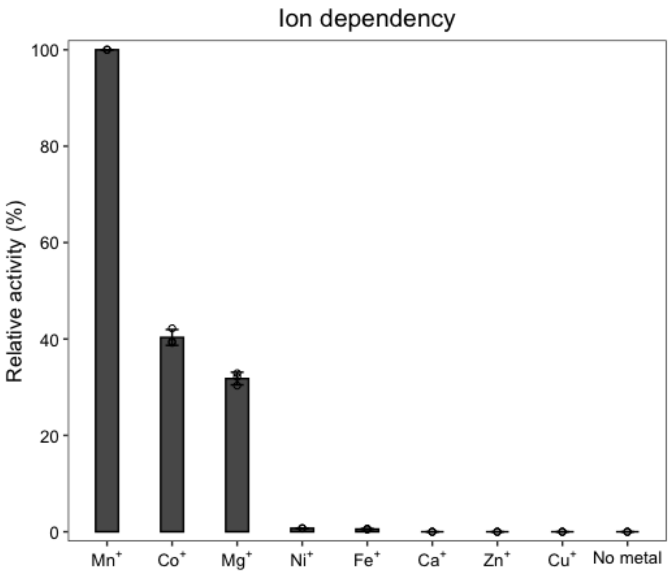

(a)

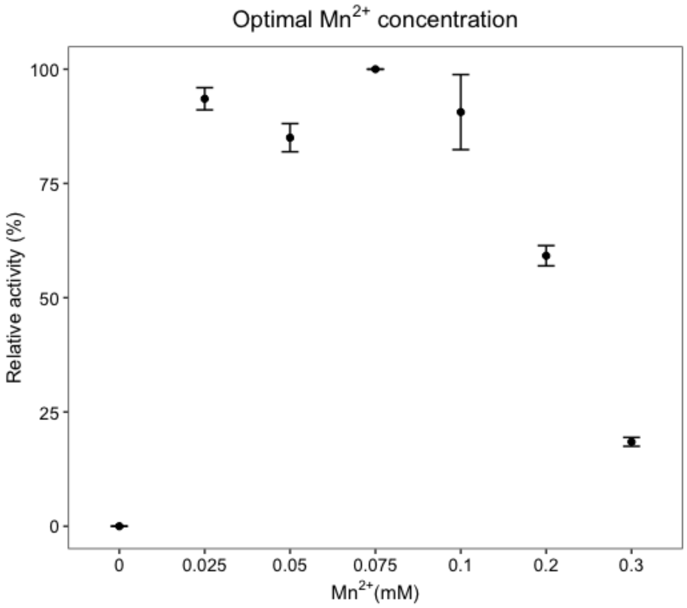

(b)

Supplementary Figure 2. Metal ion dependency of the D-xylonate dehydratase. To investigate the influence of divalent metal ions, the purified D-xylonate dehydratase (XAD) was dialysed with 50 mM HEPES/NaOH (pH 7.3, RT) containing 300 mM NaCl and 1 mM EDTA at 4°C overnight. The specific activity was determined by the TBA assay in 100 mM HEPES/NaOH (pH 7.5, 37°C) at 37°C using 10 mM D-xylonate and 3.5 µg ml^{-1} of the dialyzed XAD. The metal ion dependency was tested in absence of metal ions and in the presence of 0.075 mM $MnCl_2$, $CoCl_2$, $NiSO_4$, $FeSO_4$, $CaCl_2$, $ZnSO_4$ or $CuCl_2$ (a). Mn^{2+} showed the highest activity and to determine the optimal concentration of Mn^{2+} (b), 0-0.3 mM of $MnCl_2$ was added. The highest XAD activity (100% relative activity) was observed in the presence of .0.075 mM Mn^{2+}. All measurements were performed in triplicate (n=3), the dots in the figures are the mean values and the error bars indicate the standard deviation. Source data are provided as a Source Data file.

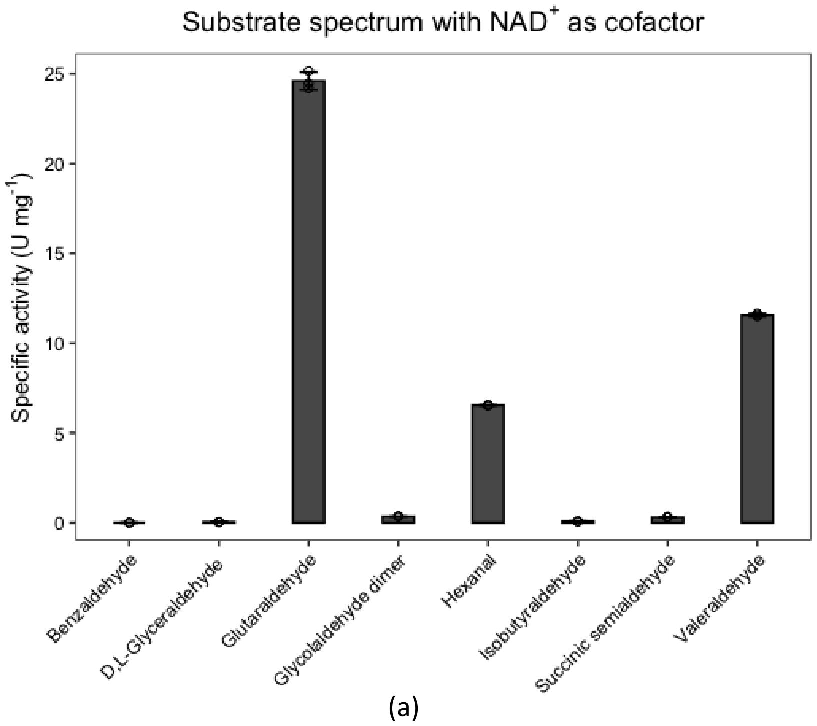

(a)

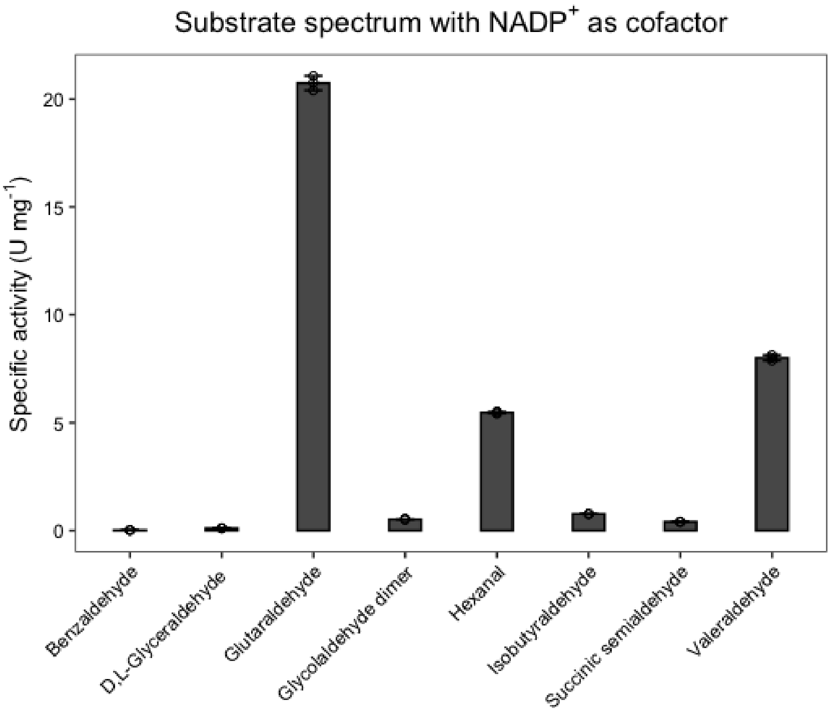

(b)

Supplementary Figure 3. Substrate spectrum of the α-ketoglutarate semialdehyde dehydrogenase. The specific activity was determined in 100 mM HEPES/NaOH (pH 7.5, 37°C) at 37°C using 0.9-17.5 µg α-ketoglutarate semialdehyde dehydrogenase (KGSADH), 5 mM D,L-glyceraldehyde, glycolaldehyde dimer, benzaldehyde, valeraldehyde, hexanal or isobutyraldehyde with 5 mM NAD^+ (a) or $NADP^+$ (b) as cofactor. All measurements were performed in triplicate (n=3), the dots in the figures are the mean values and the error bars indicate the standard deviation. Source data are provided as a Source Data file.

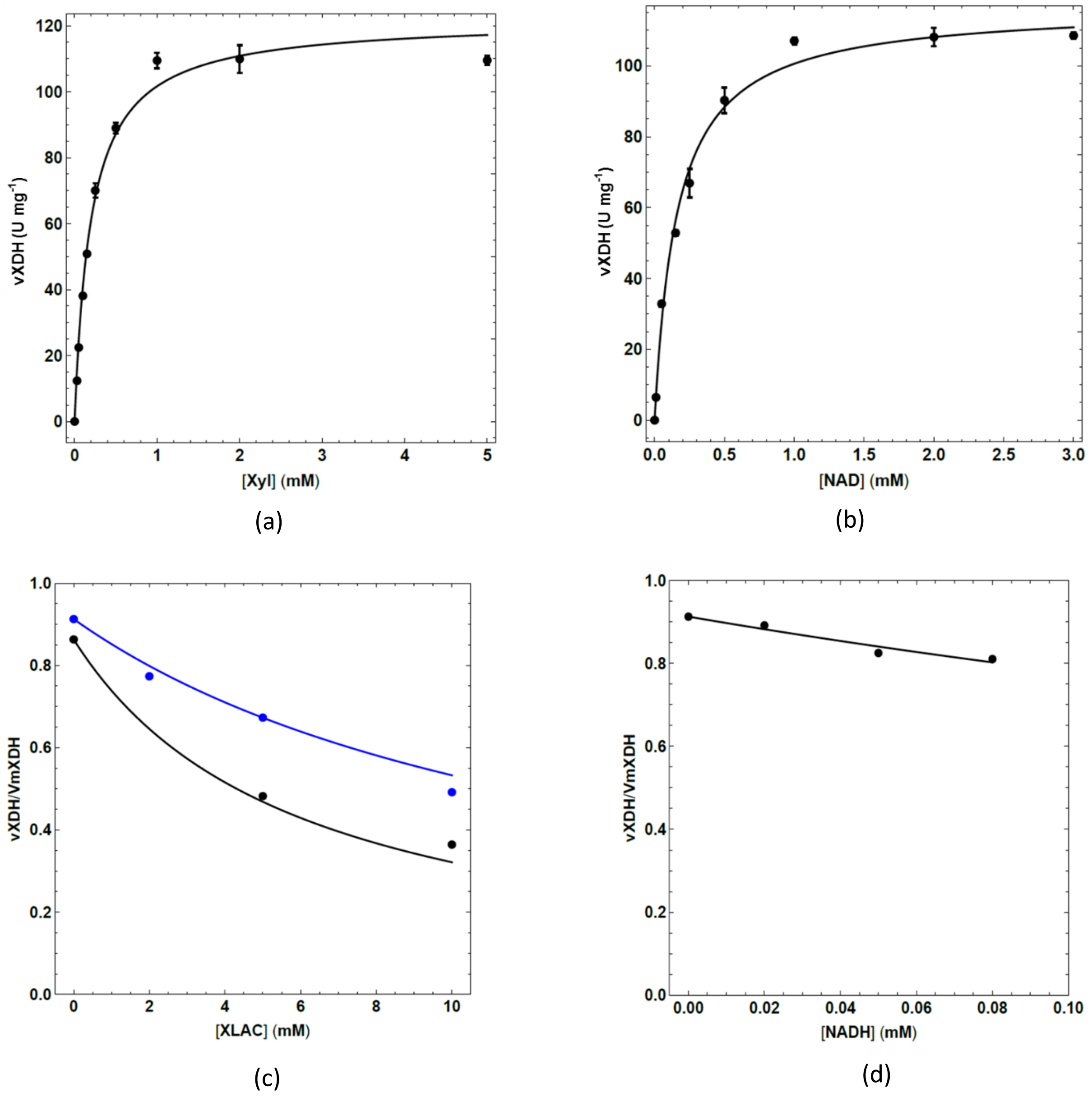

Supplementary Figure 4. Characterization of D-xylose dehydrogenase. The D-xylose dehydrogenase (XDH) activity as a function of D-xylose and NAD^+, and product inhibition by D-xylonolactone and NADH is shown. Initial rate kinetics were measured for the purified XDH (0.136 µg ml^{-1}) by following NADH formation at various concentrations of D-xylose (Xyl) (panel (a), with 5 mM NAD^+) and at various concentrations of NAD^+ (panel (b), with 2 mM Xyl). The data without added product were fitted to Eq. 1; inhibition by D-xylonolactone (XLAC) (panel (c), measured with 3 mM NAD^+ and 2 mM Xyl (shown in black) or 5 mM Xyl (shown in blue)), and inhibition by NADH (panel (d), measured with 5 mM Xyl and 3 mM NAD^+), was fitted separately, and expressed as fraction of maximal activity (panel (c) and (d)). Measurements in (a) and (b) were performed in triplicate (n=3), the dots in the figures are the mean values and the error bars indicate the standard deviation. Source data are provided as a Source Data file. It also can be accessed via http://doi.org/10.15490/FAIRDOMHUB.1.ASSAY.968.1.

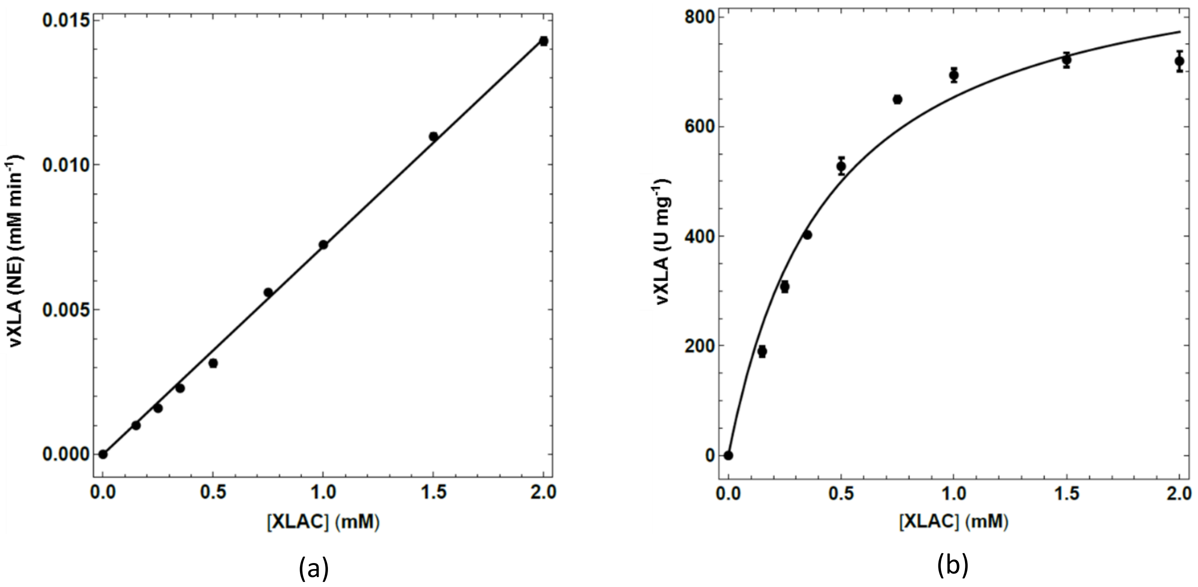

Supplementary Figure 5. Enzymatic and non-enzymatic xylonolactonase activity as a function of D-xylonolactone. Initial rate kinetics were measured for the non-enzymatic reaction and for purified xylonolactonase (XLA) (0.0069 µg ml^{-1}) by following NADH formation at various concentrations of D-xylonolactone (XLAC). The reaction was linked to XAD, KDXD and KGSADH. The lines are the fitted equations 2 (panel (a)), and 3 (panel (b)) with parameter values listed in Supplementary Table 4. All measurements were performed in triplicate (n=3), the dots in the figures are the mean values and the error bars indicate the standard deviation. Source data are provided as a Source Data file. It also can be accessed via http://doi.org/10.15490/FAIRDOMHUB.1.ASSAY.969.1.

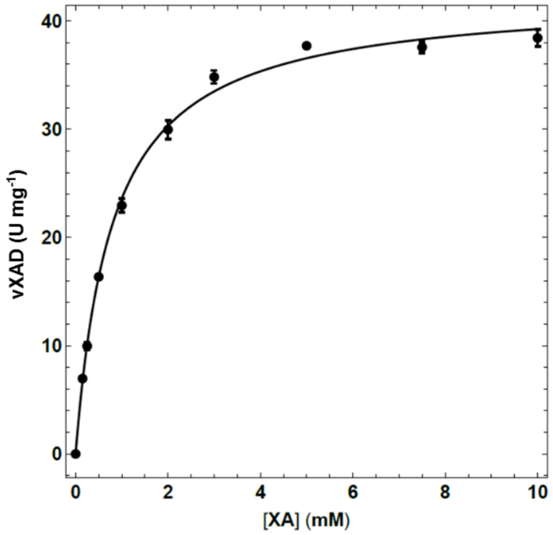

Supplementary Figure 6. D-xylonate dehydratase activity as a function of D-xylonate. Initial rate kinetics were measured for purified D-xylonate dehydratase (XAD) (3.29 µg ml^{-1}) using an off-line colorimetric assay. XA: D-xylonate. The line is the fitted equation 4 with parameter values listed in Table 5. Measurements were performed in triplicate (n=3), the dots in the figures are the mean values and the error bars indicate the standard deviation. Source data are provided as a Source Data file. It also can be accessed via http://doi.org/10.15490/FAIRDOMHUB.1.ASSAY.970.1.

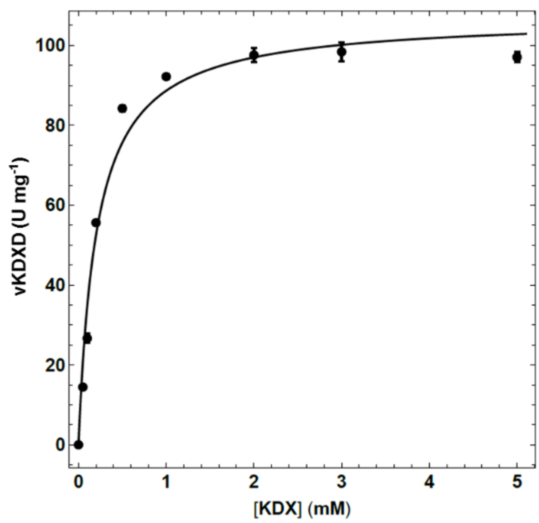

(a)

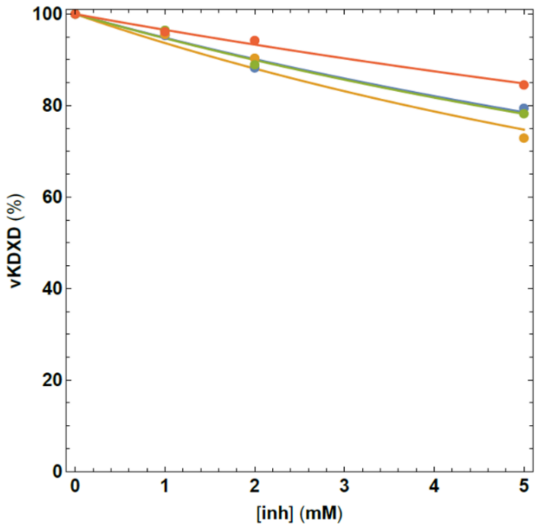

(b)

Supplementary Figure 7. Characterization of 2-keto-3-deoxy-D-xylonate dehydratase. The activity of the 2-keto-3-deoxy-D-xylonate dehydratase (KDXD) as a function of 2-keto-3-deoxy-D-xylonate, and the effect of inhibitors is shown. Initial rate kinetics were measured for purified KDXD (0.189 µg ml^{-1}) by following NADH formation at various concentrations of 2-keto-3-deoxy-D-xylonate (KDX), in a KGSADH linked assay. The lines in panel (a) are the fitted equation 5 with parameter values listed in Supplementary Table 6. The inhibitory effect of lactate (shown in red), pyruvate (shown in green), *α*-ketoglutarate (shown in yellow), and D-xylonate (shown in blue), measured at 2 mM KDX concentration, are indicated in panel (b). Measurements in (a) were performed in triplicate (n=3), the dots in the figures are the mean values and the error bars indicate the standard deviation. Source data are provided as a Source Data file. It also can be accessed via http://doi.org/10.15490/FAIRDOMHUB.1.ASSAY.971.1.

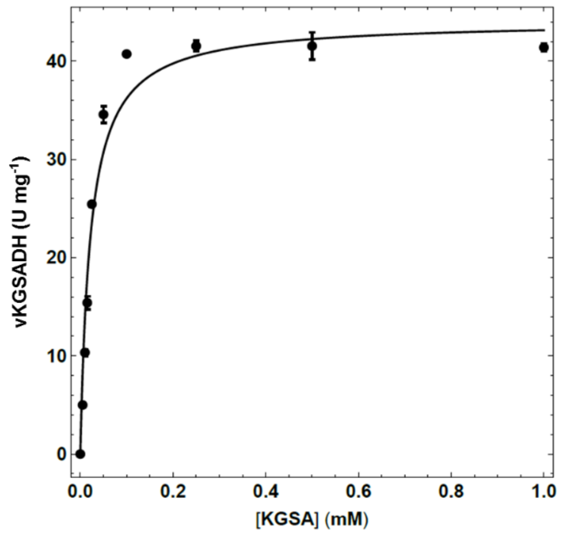

vKGSADH (U mg-1)
[KGSA] (mM)
0
10
20
30
40
0.0
0.2
0.4
0.6
0.8
1.0

(a)

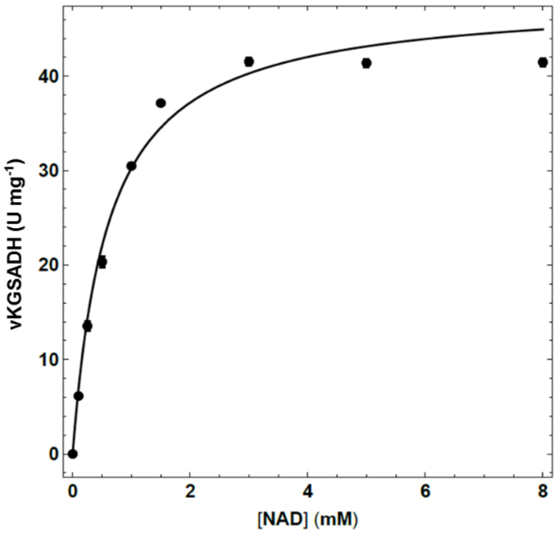

vKGSADH (U mg-1)
[NAD] (mM)
0
10
20
30
40
0
2
4
6
8

(b)

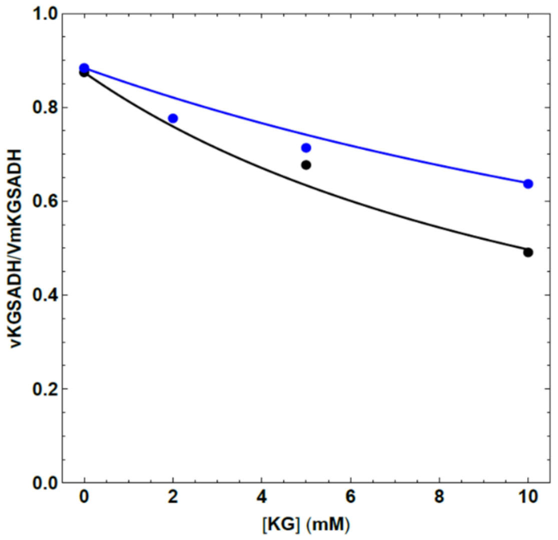

vKGSADH/VmKGSADH
[KG] (mM)
0.0
0.2
0.4
0.6
0.8
1.0
0
2
4
6
8
10

(c)

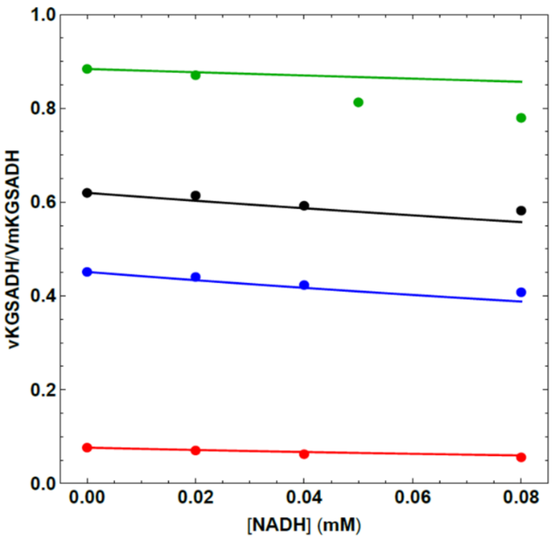

vKGSADH/VmKGSADH
[NADH] (mM)
0.0
0.2
0.4
0.6
0.8
1.0
0.00
0.02
0.04
0.06
0.08

(d)

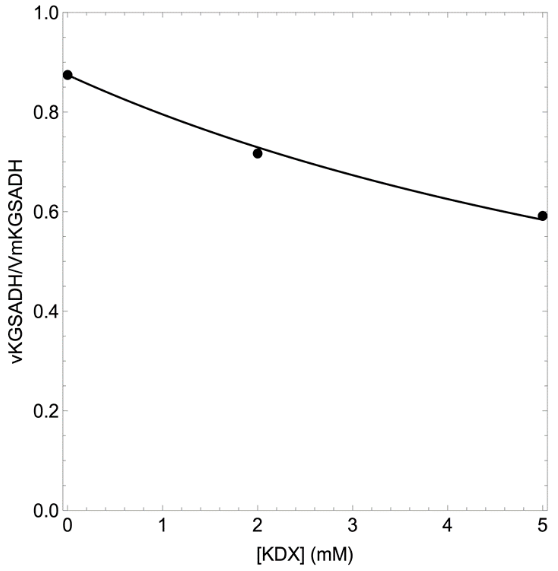

vKGSADH/VmKGSADH
[KDX] (mM)
0.0
0.2
0.4
0.6
0.8
1.0
0
1
2
3
4
5

(e)

Supplementary Figure 8. Characterization of α-ketoglutarate semialdehyde dehydrogenase. The activity of the α-ketoglutarate semialdehyde dehydrogenase (KGSADH) as a function of α-ketoglutarate semialdehyde (KGSA) and NAD^+, and product inhibition by α-ketoglutarate (KG) and NADH is shown. Initial rate kinetics were measured for purified KGSADH (0.1324 µg ml^{-1}) by following NADH formation at various concentrations of KGSA (with 5 mM NAD^+, panel (a)) and at various concentrations of NAD^+ (with 1 mM KGSA, panel (b)). Product inhibition for KG was measured with 5 mM NAD^+, and either 1 mM KGSA (shown in black) or 2 mM KGSA (shown in blue) in panel (c). Product inhibition for NADH was measured with 5 mM (shown in green), 1 mM (shown in black), 0.5 mM (shown in blue), or 0.05 mM (shown in red) NAD^+, and 1 mM KGSA in panel (d). Inhibition by 2-keto-3-deoxy-D-xylonate was measured with 5 mM NAD^+ and 1 mM KGSA in panel (e). The substrate data set was fitted to Eq. 6, and product inhibition was fitted separately, resulting in parameter values listed in Supplementary Table 7. For the inhibition plots the activity is expressed as fraction of the V_M of the enzyme. Measurements in (a) and (b) were performed in triplicate (n=3), the dots in the figures are the mean values and the error bars indicate the standard deviation. Source data are provided as a Source Data file. It also can be accessed via http://doi.org/10.15490/FAIRDOMHUB.1.ASSAY.972.1.

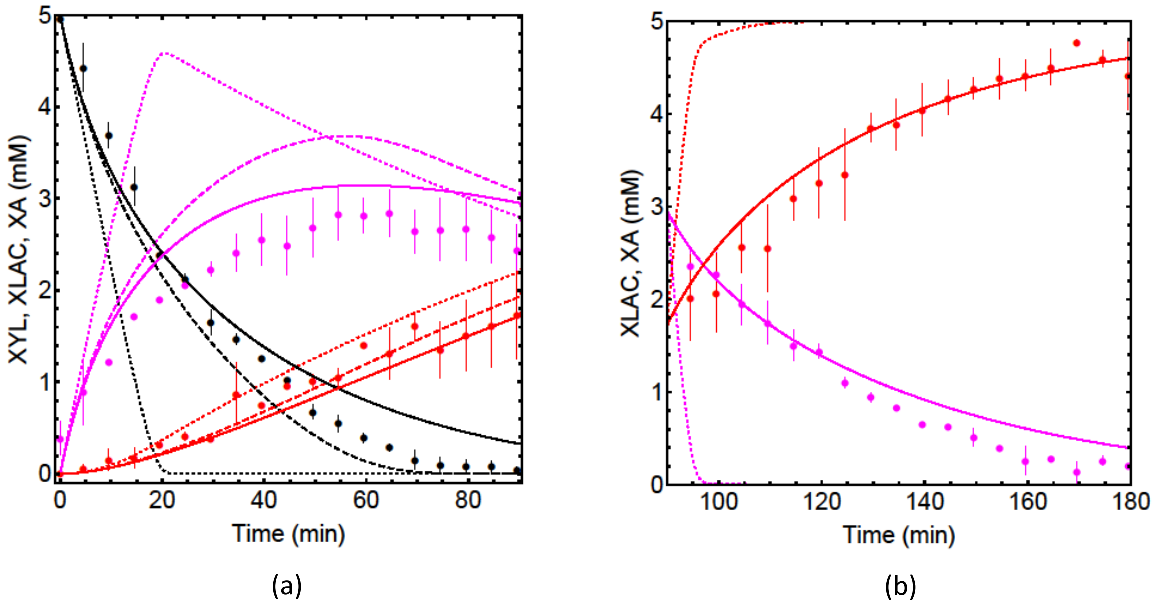

Supplementary Figure 9. Progress curves for D-xylose dehydrogenase and xylonolactonase. Conversion of 5 mM D-xylose (XYL) (shown in black) by D-xylose dehydrogenase (XDH) (2.5 µg ml^{-1}) was followed over time with NMR (panel (a). D-xylonolactone (XLAC) (shown in pink) formed by XDH, was non-enzymatically degraded to D-xylonate (XA) (shown in red). At t = 90 min, xylonolactonase (XLA) (0.8 µg ml^{-1}) was added to the incubation, and the conversion of XLAC to XA was followed in panel (b). For panel (a), a mathematical model based on Eqs. 1 and 2 was used, for panel (b), Eq. 3 was additionally added to the model. Model simulations are shown in solid lines for the full rate equation. In panel (a), dotted lines are for predicted conversion without inhibition, and dashed lines are for predicted conversion with NADH inhibition but no XLAC inhibition. In panel (b), dashed lines are for predicted conversion without product inhibition. All simulations are shown with corresponding colors to the experimental symbols. The dots in the figures are the mean values of the results from two independent experiments (n=2) and the error bars indicate the standard error of the mean. Source data are provided as a Source Data file. It also can be accessed via http://doi.org/10.15490/FAIRDOMHUB.1.ASSAY.978.2 and http://doi.org/10.15490/FAIRDOMHUB.1.ASSAY.979.2.

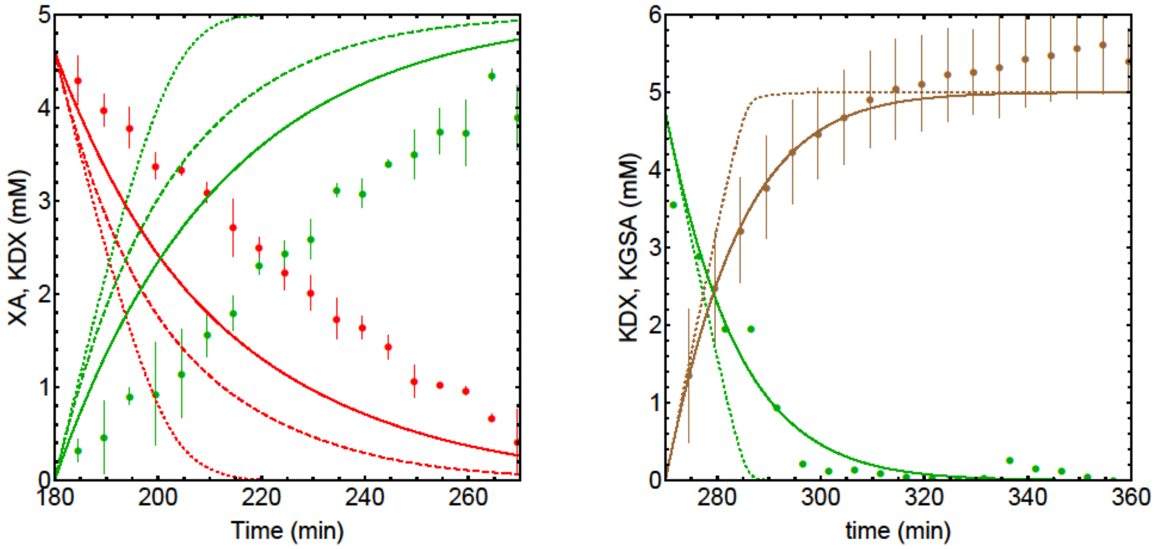

(a) (b)

Supplementary Figure 10. D-xylonate dehydratase and 2-keto-3-deoxy-D-xylonate dehydratase progress curves. Conversion of 5 mM D-xylonate (XA) (formed in a XDH, XLA incubation, see Supplementary Fig. 9, shown in red) to 2-keto-3-deoxy-D-xylonate (KDX) (shown in green), after addition of D-xylonate dehydratase (XAD) (6.5 µg ml^{-1}) at 180 min, was followed over time with NMR (panel (a). At t = 270 min, 2-keto-3-deoxy-D-xylonate dehydratase (KDXD) (3.3 µg ml^{-1}) was added to the incubation, and the conversion of KDX to *α*-ketoglutarate semialdehyde (KGSA) was followed in panel (b). For panel (a), a mathematical model based on Eqs. 1, 2, 3, and 4 was used, for panel (b), Eq. 5 was additionally added to the model. Model simulations are shown in solid lines for the full rate equation. In panel (a), dotted lines are for predicted conversion without inhibition, and dashed lines are for predicted conversion with NADH inhibition but no KDX inhibition. In panel (b), dotted lines are for predicted conversion without product inhibition. All simulations are shown with corresponding colors to the experimental symbols. The dots in the figures are the mean values of the results from two independent experiments (n=2) and the error bars indicate the standard error of the mean. Source data are provided as a Source Data file. It also can be accessed via http://doi.org/10.15490/FAIRDOMHUB.1.ASSAY.980.2 and http://doi.org/10.15490/FAIRDOMHUB.1.ASSAY.981.2.

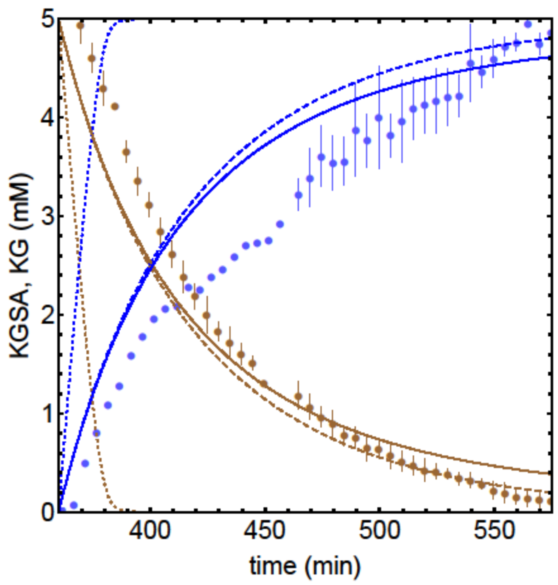

Supplementary Figure 11. α-ketoglutarate semialdehyde dehydrogenase progress curve. Conversion of 5 mM *α*-ketoglutarate semialdehyde (KGSA) (formed in a XDH, XLA, XAD, KDXD incubations, see Supplementary Fig. 9 and 10, and shown in brown), to α-ketoglutarate (KG) (shown in blue), after addition of α-ketoglutarate semialdehyde dehydrogenase (KGSADH) (6.6 µg ml^{-1}) at 360 min, was followed over time with NMR. Data shown are mean values with standard error for two independent experiments. A mathematical model based on Eqs. 1, 2, 3, 4, 5 and 6 was used to describe the experiment. Model simulations are shown in solid lines for the full rate equation, dotted lines are for predicted conversion without inhibition, and dashed lines are for predicted conversion with NADH inhibition but no other products inhibition. All simulations are shown with corresponding colors to the experimental symbols. The dots in the figure are the mean values of the results from two independent experiments (n=2) and the error bars indicate the standard error of the mean. Source data are provided as a Source Data file. It also can be accessed via http://doi.org/10.15490/FAIRDOMHUB.1.ASSAY.982.2.

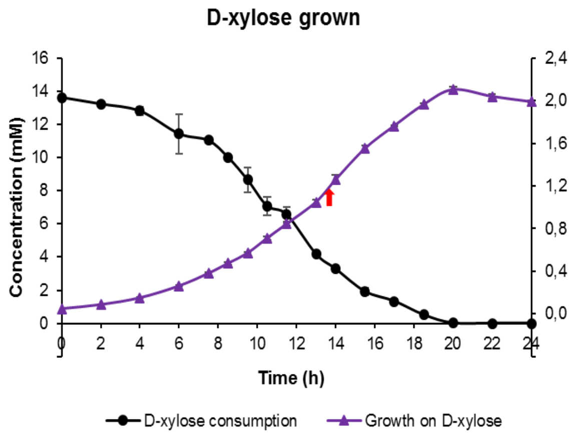

(a)

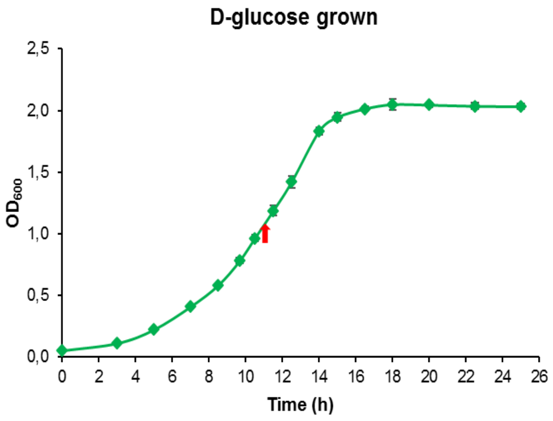

(b)

Supplementary Figure 12. Cell growth of *C. crescentus* NA1000. *C. crescentus* NA1000 was grown on minimum medium supplemented with 0.2% D-xylose (a) or D-glucose (b). The consumption of D-xylose in the D-xylose grown cultures were monitored. The red arrows in the growth curves indicate where the cells were collected for enzyme activity determination and D-xylose conversion experiments. Cultures were grown in triplicate (n=3), the dots in the figures are the mean values and the error bars indicate the standard deviation. Source data are provided as a Source Data file.

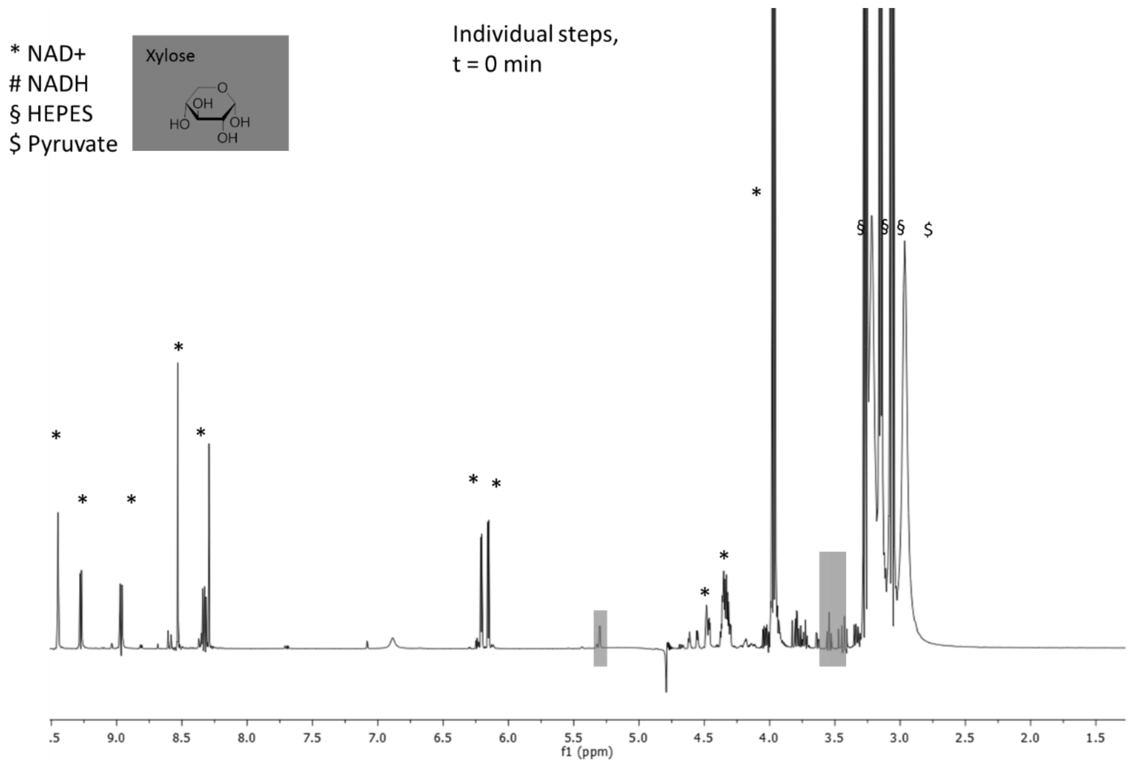

Supplementary Figure 13. 1H-NMR spectrum of D-xylose by 1H-NMR. The spectrum above is taken from the sequential enzyme cascade analysis.

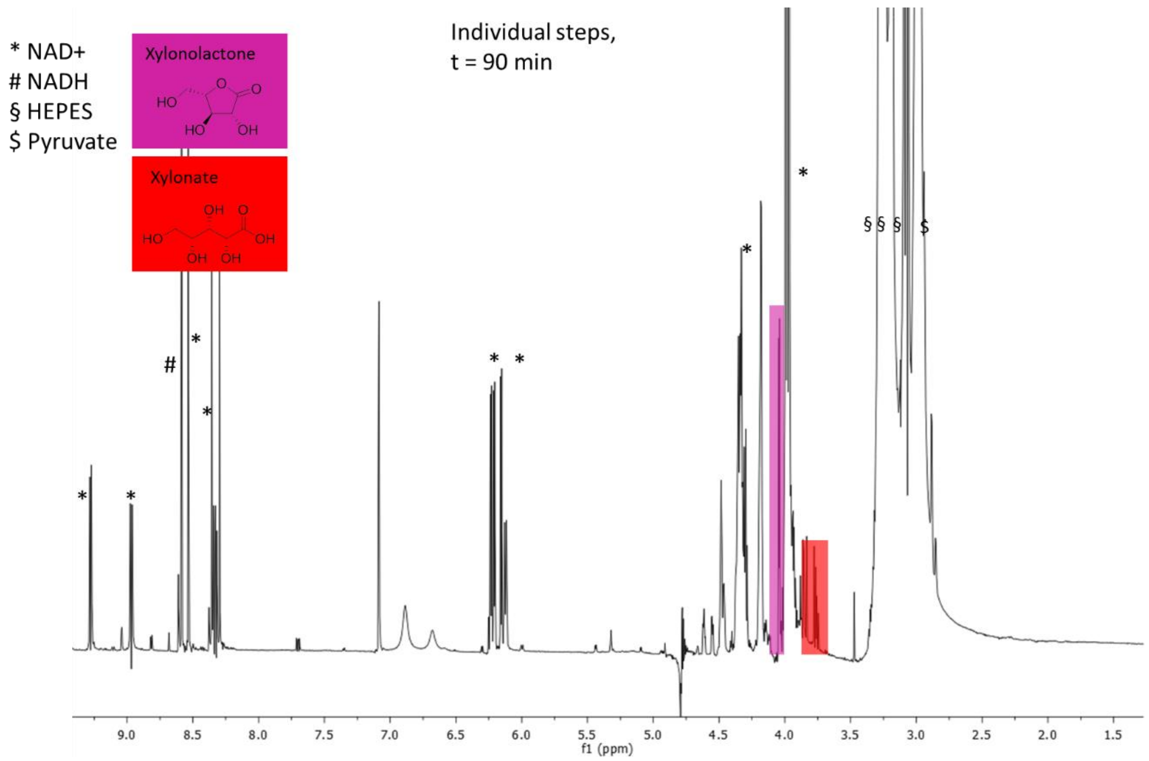

Supplementary Figure 14. 1H-NMR spectrum of xylonolactone and D-xylonate by 1H-NMR. The spectrum above is taken from the sequential enzyme cascade analysis.

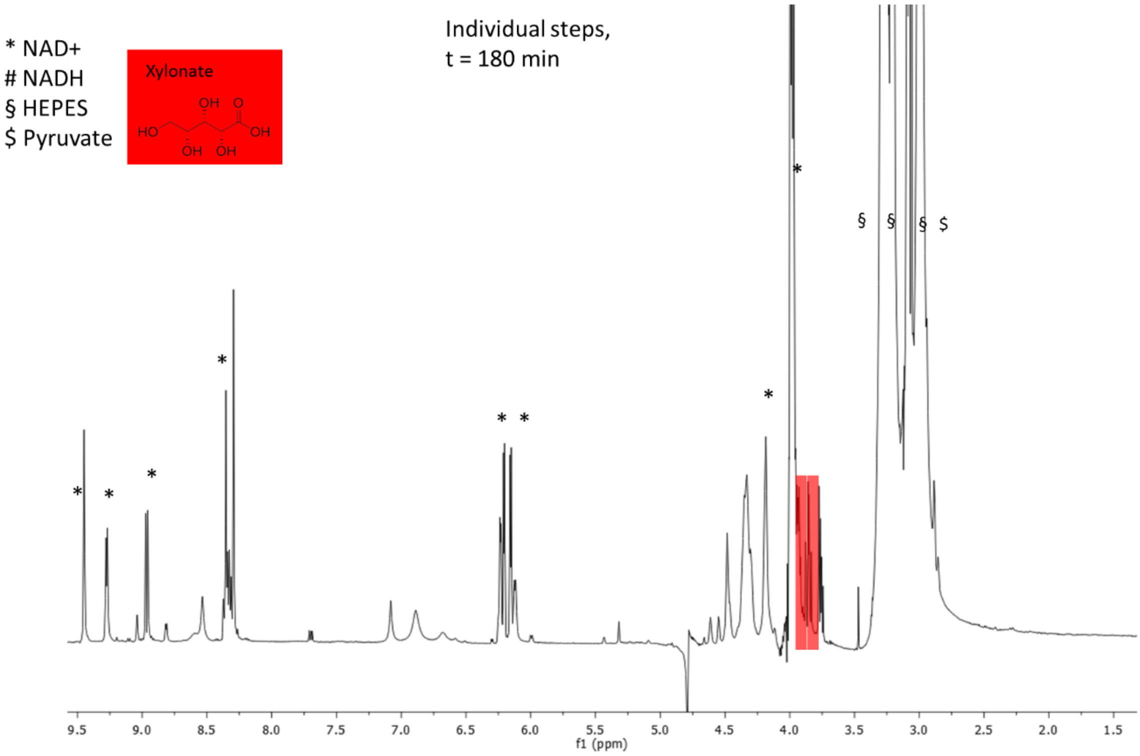

Supplementary Figure 15. ^{1}H-NMR spectrum of D-xylonate by ^{1}H-NMR. The spectrum above is taken from the sequential enzyme cascade.

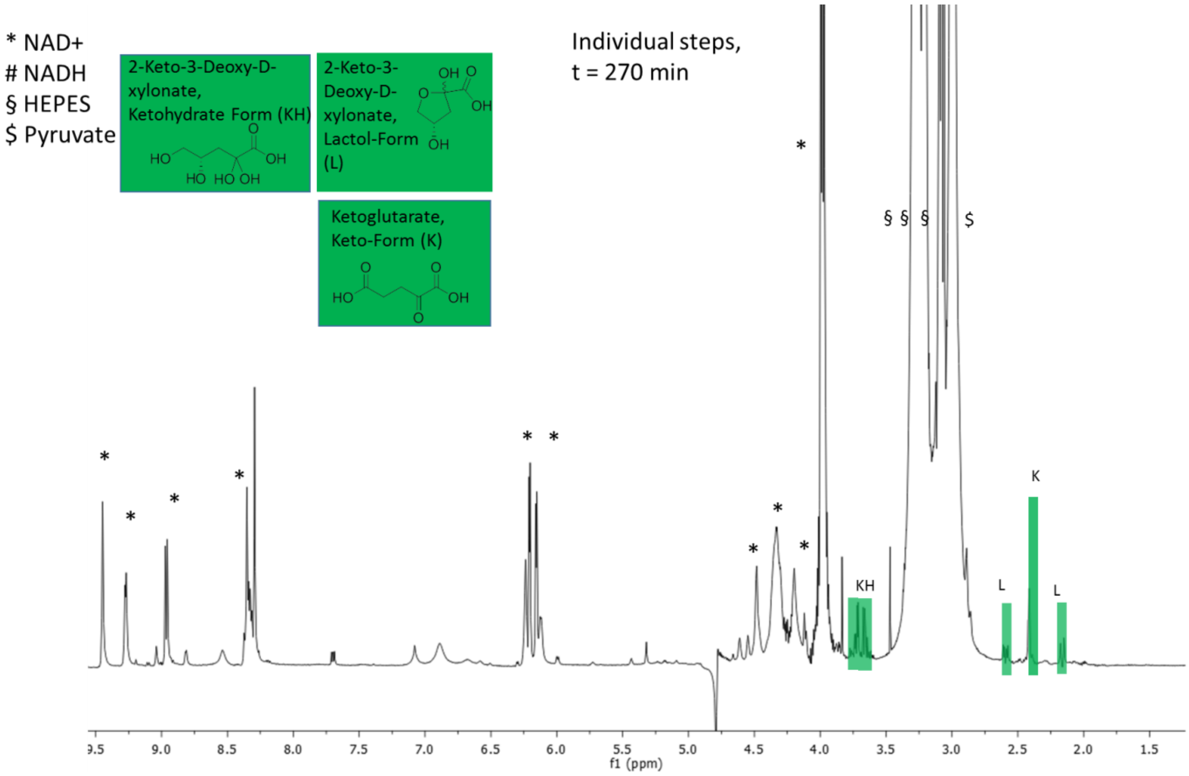

Supplementary Figure 16. ^{1}H-NMR spectrum of 2-keto-3-deoxy-D-xylonate by ^{1}H-NMR. The mixture contains the ketohydrate-, keto- and lactol-form. The spectrum above is taken from the sequential enzyme cascade analysis.

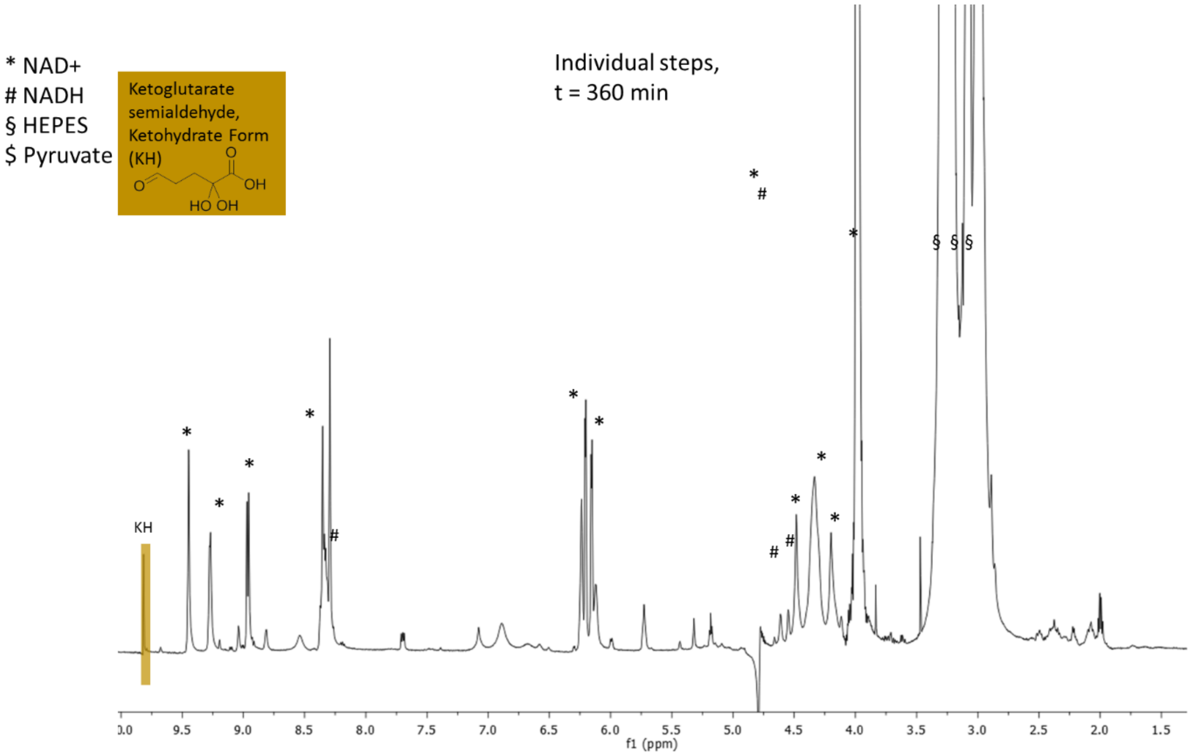

Supplementary Figure 17. ^{1}H-NMR spectrum of α-ketoglutarate semialdehyde by ^{1}H-NMR. The spectrum above is taken from the sequential enzyme cascade analysis.

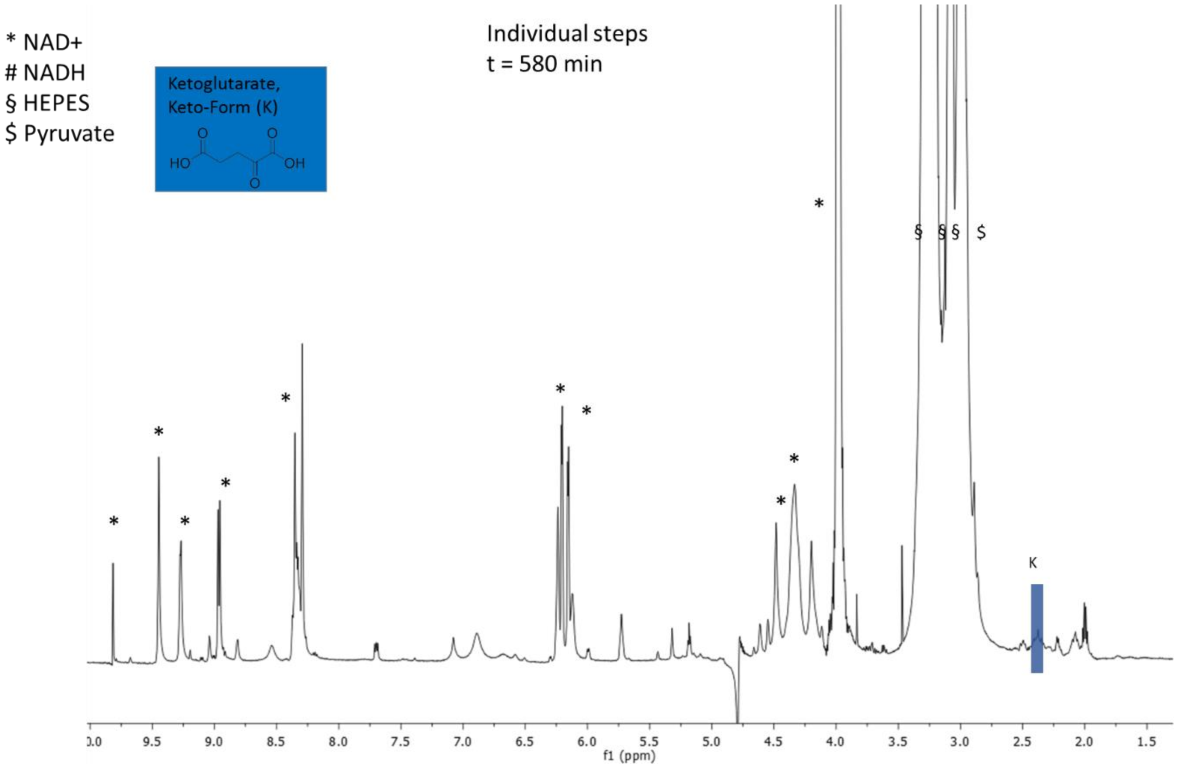

Supplementary Figure 18. ^{1}H-NMR spectrum of α-ketoglutarate by ^{1}H-NMR. The spectrum above is taken from the sequential enzyme cascade analysis.

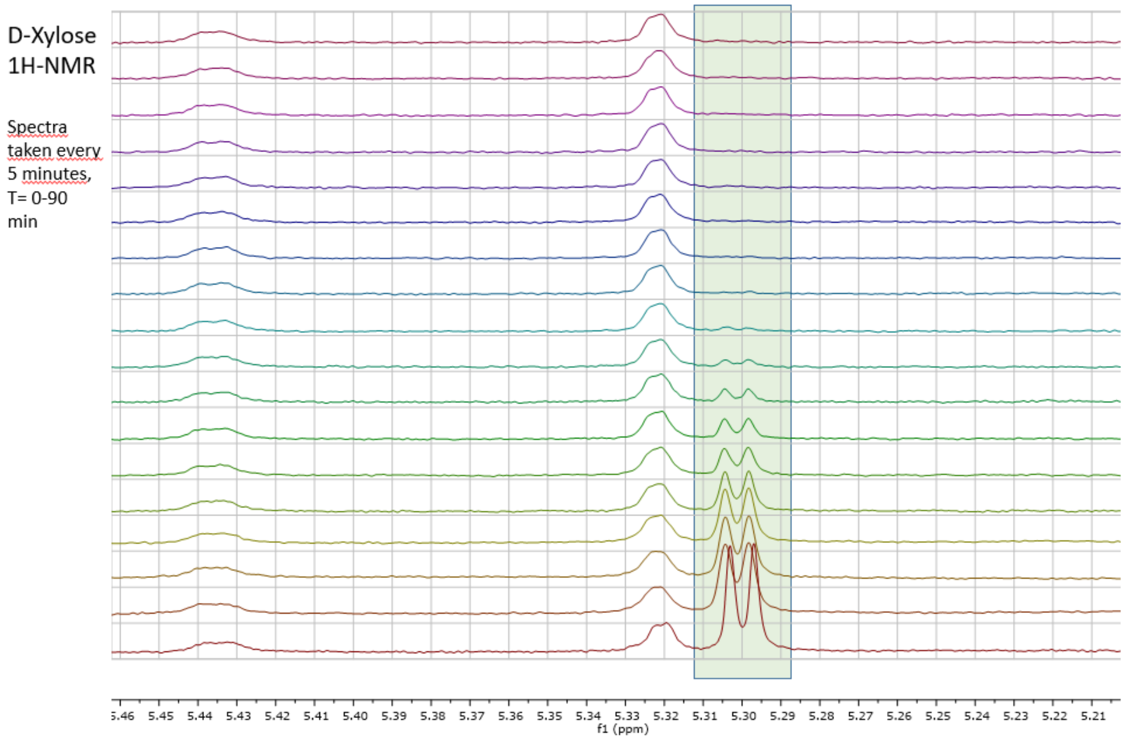

Supplementary Figure 19. Evolution of D-xylose concentration by ^{1}H-NMR. Stacked ^{1}H-NMR spectra following the decreasing signal for D-xylose (see highlighted area).

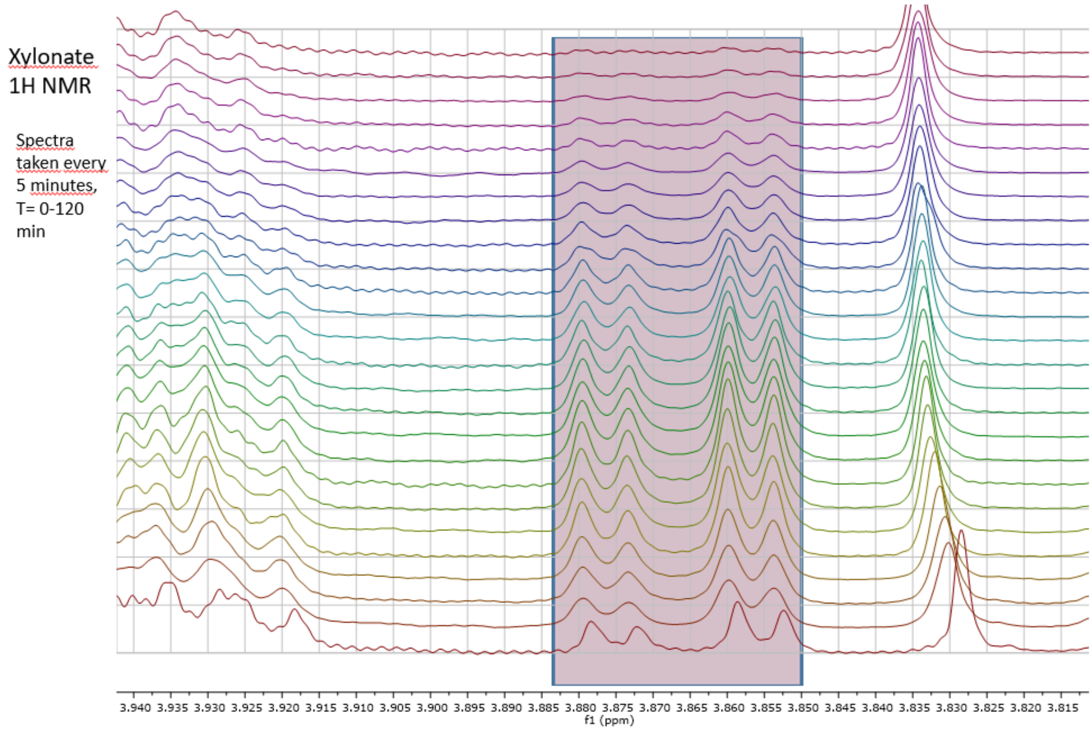

Supplementary Figure 20. Evolution of D-xylonate concentration by ^{1}H-NMR. Stacked ^{1}H-NMR spectra following the changing signal for D-xylonate (see highlighted area).

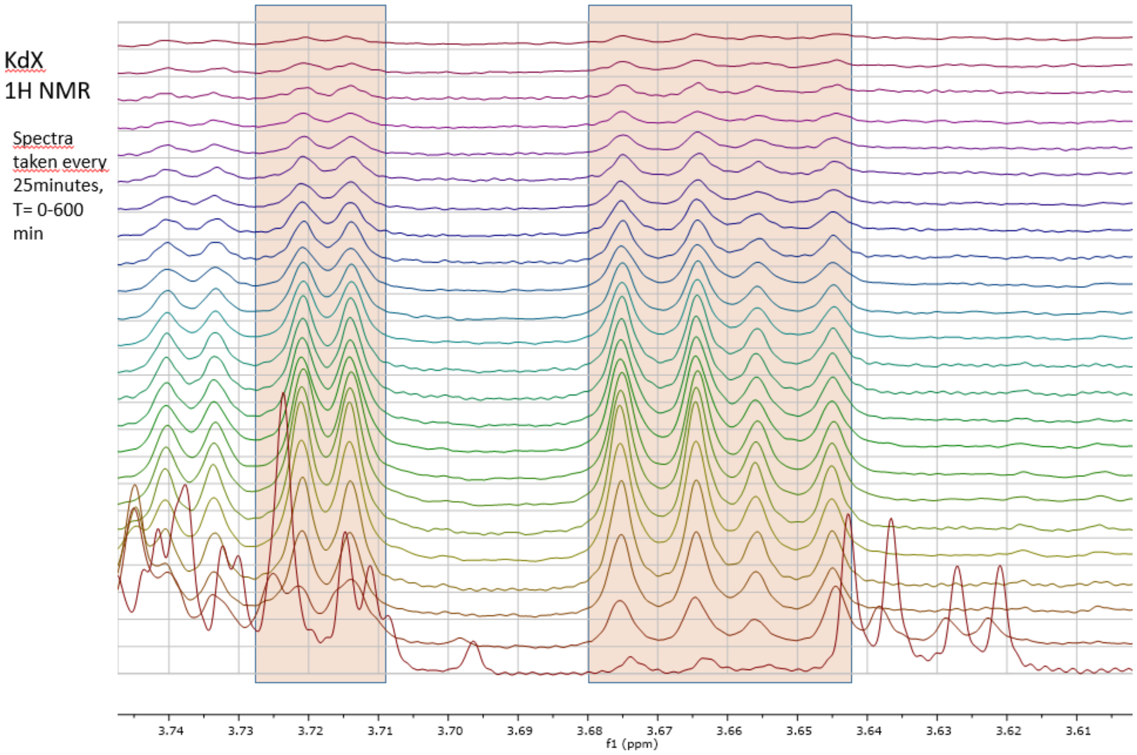

Supplementary Figure 21. Evolution of 2-keto-3-deoxy-D-xylonate concentration by ^{1}H-NMR. Stacked ^{1}H-NMR spectra following the changing signal for 2-keto-3-deoxy-D-xylonate (see highlighted area).

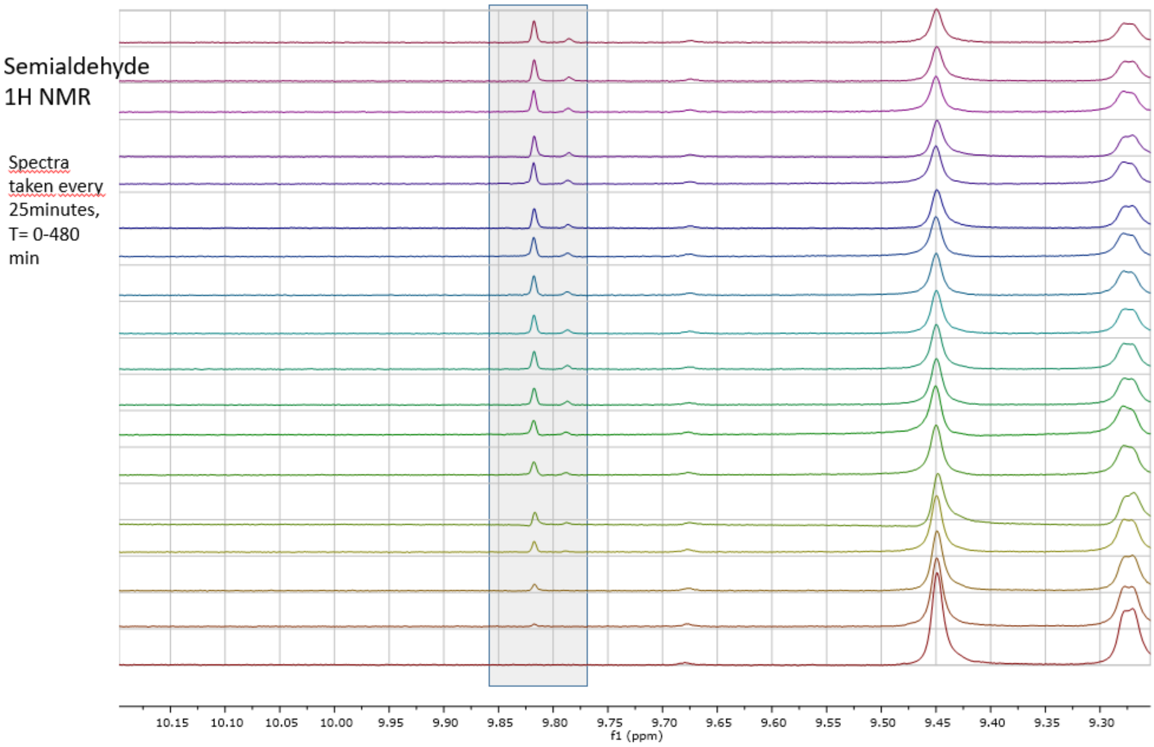

Supplementary Figure 22. Evolution of α-ketoglutarate semialdehyde concentration by ^{1}H-NMR. Stacked ^{1}H-NMR spectra following the increasing signal for α-ketoglutarate semialdehyde (see highlighted area).

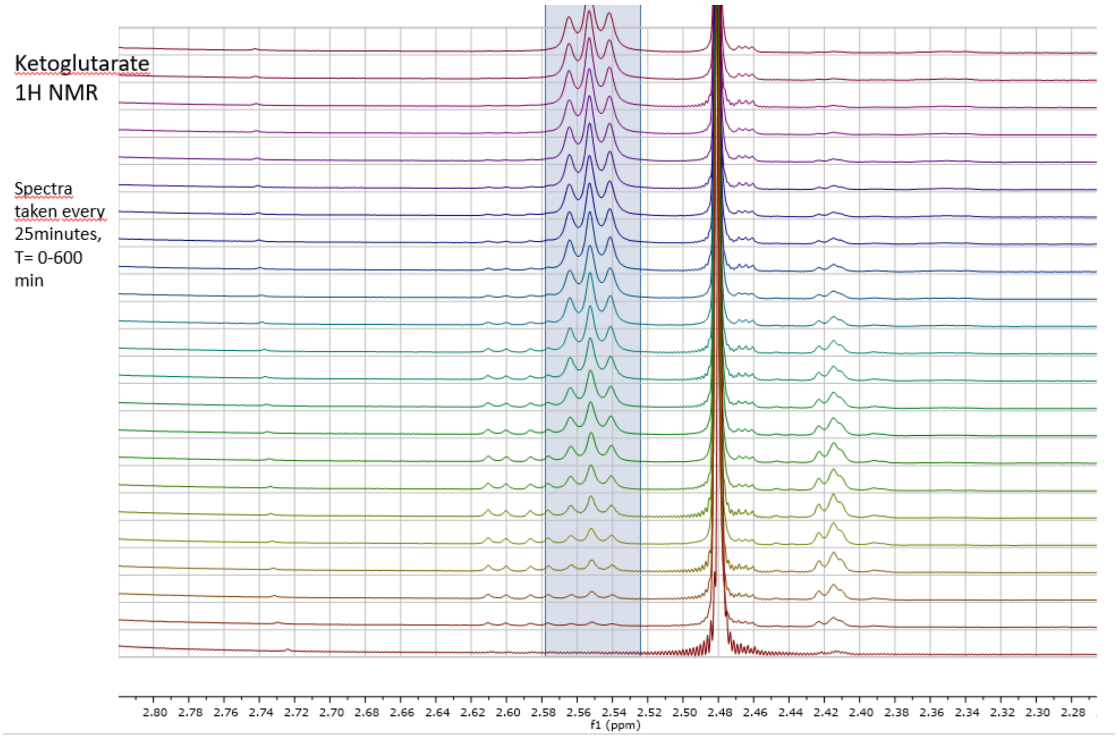

Supplementary Figure 23. Evolution of α-ketoglutarate concentration by ^{1}H-NMR. Stacked ^{1}H-NMR spectra following the increasing signal for α-ketoglutarate (see highlighted area).

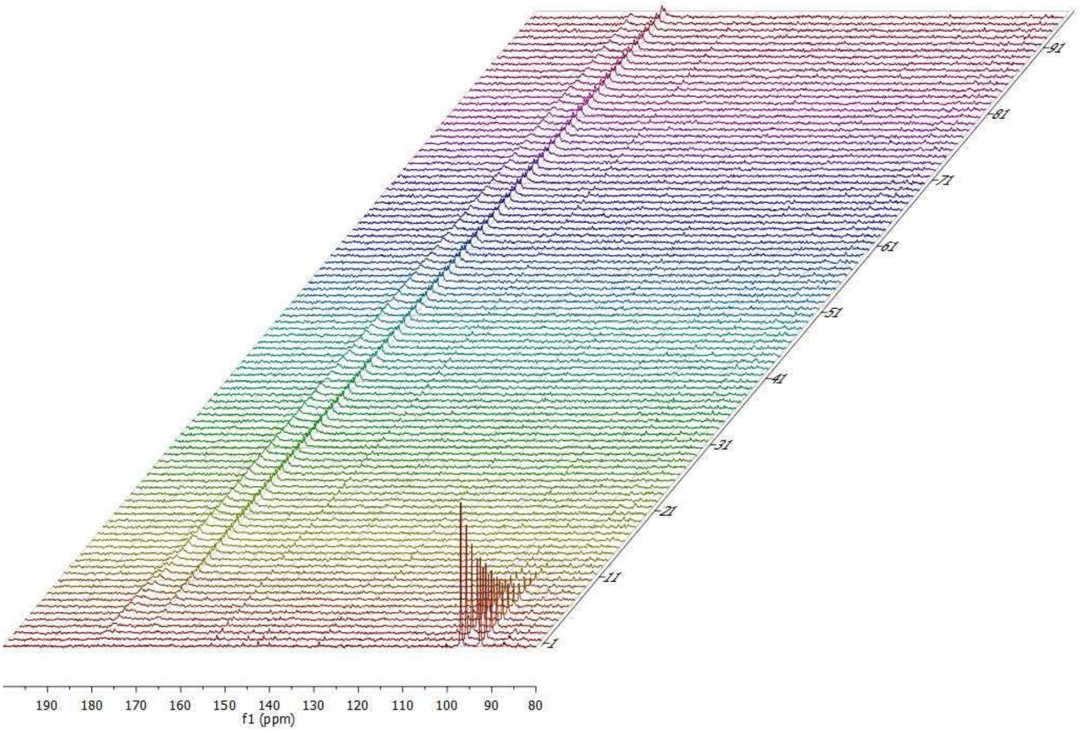

Supplementary Figure 24. Overall reaction progress of the reference state followed by ^{13}C-NMR . Stacked ^{13}C- NMR spectra highlighting the decrease of signal intensity for D-xylose (at 100ppm) and the increase of signal intensity for xylonolactone (at 180ppm), D-xylonate (at 178ppm), 2-keto-3-deoxy-D-xylonate (at 170ppm) and α-ketoglutarate (at 171ppm) in the reference one-pot enzyme cascade analysis.

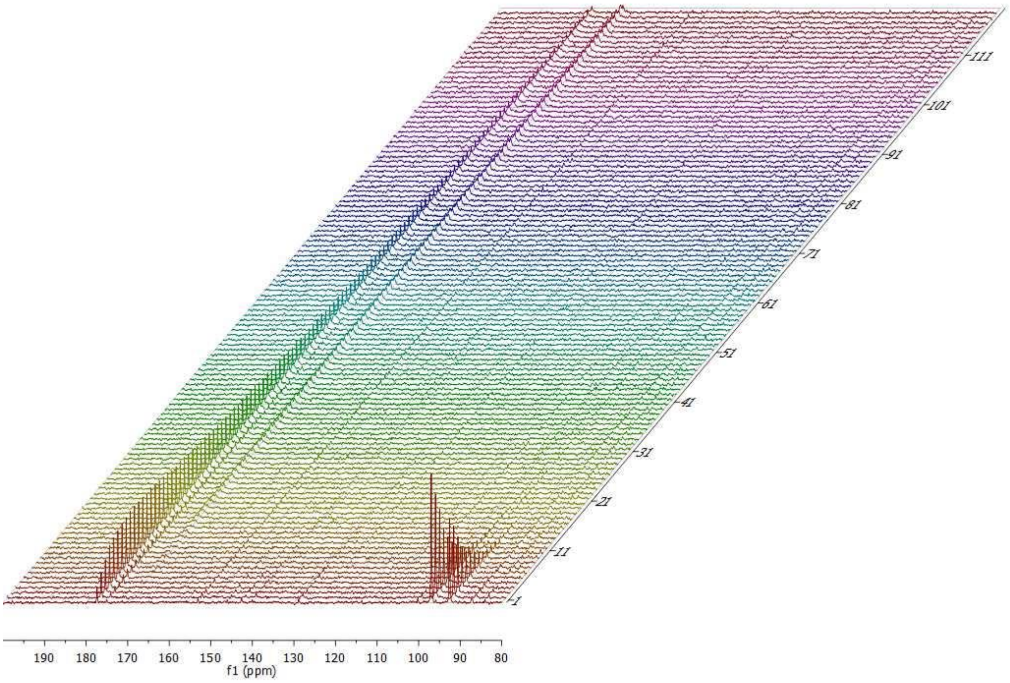

Supplementary Figure 25. Overall reaction progress of the cascade without xylonolactonase followed by ^{13}C-NMR. Stacked ^{13}C-NMR spectra highlighting the decrease of signal intensity for D-xylose (at 100ppm) and the increase of signal intensity for xylonolactone (at 180ppm), D-xylonate (at 178ppm), 2-keto-3-deoxy-D-xylonate (at 170ppm) and α-ketoglutarate (at 171ppm) in the one-pot enzyme cascade analysis without xylonolactonase.

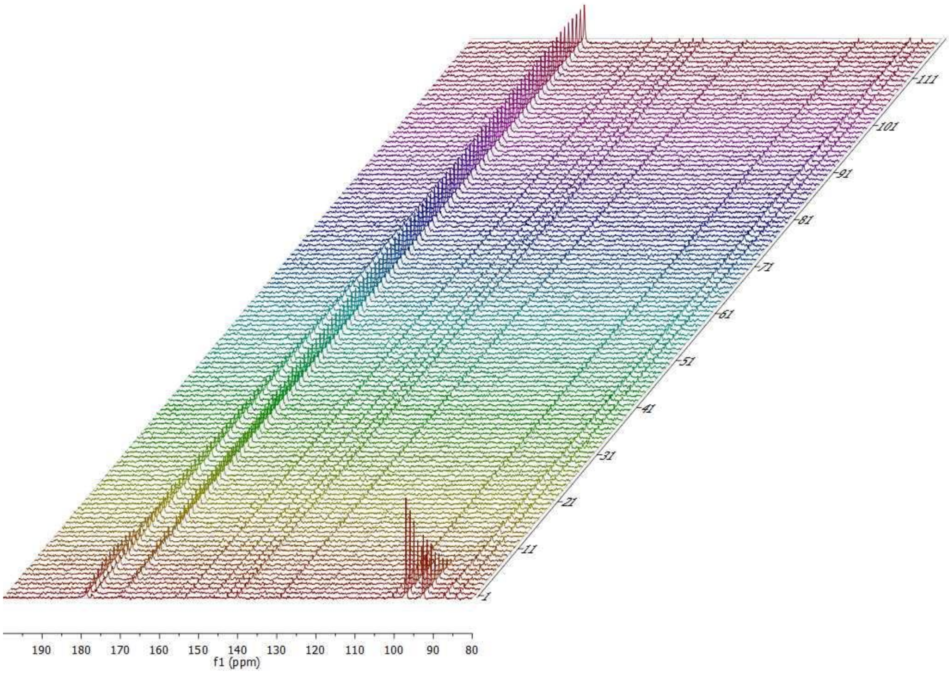

Supplementary Figure 26. Overall reaction progress of the cascade with NAD^+ recycling followed by ^{13}C-NMR. Stacked ^{13}C- NMR spectra highlighting the decrease of signal intensity for D-xylose (at 100ppm) and the increase of signal intensity for Xylonolactone (at 180ppm), D-xylonate (at 178ppm), 2-keto-3-deoxy-D-xylonate (at 170ppm) and α-ketoglutarate (at 171ppm) in the one-pot enzyme cascade analysis employing NAD^+ recycling.

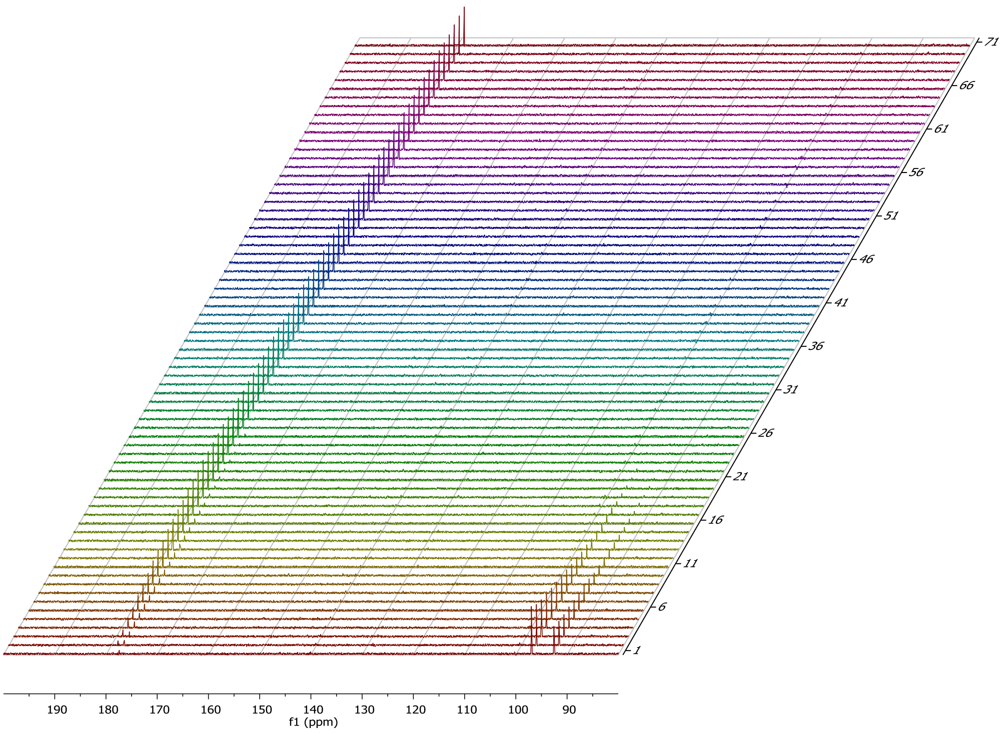

Supplementary Figure 27. Overall reaction progress of the optimized cascade followed by ^{13}C-NMR. Stacked ^{13}C- NMR spectra highlighting the decrease of signal intensity for D-xylose (at 100ppm) and the increase of signal intensity for xylonolactone (at 180ppm), D-xylonate (at 178ppm), 2-keto-3-deoxy-D-xylonate (at 170ppm) and α-ketoglutarate (at 171ppm) in the optimized one-pot enzyme cascade analysis.

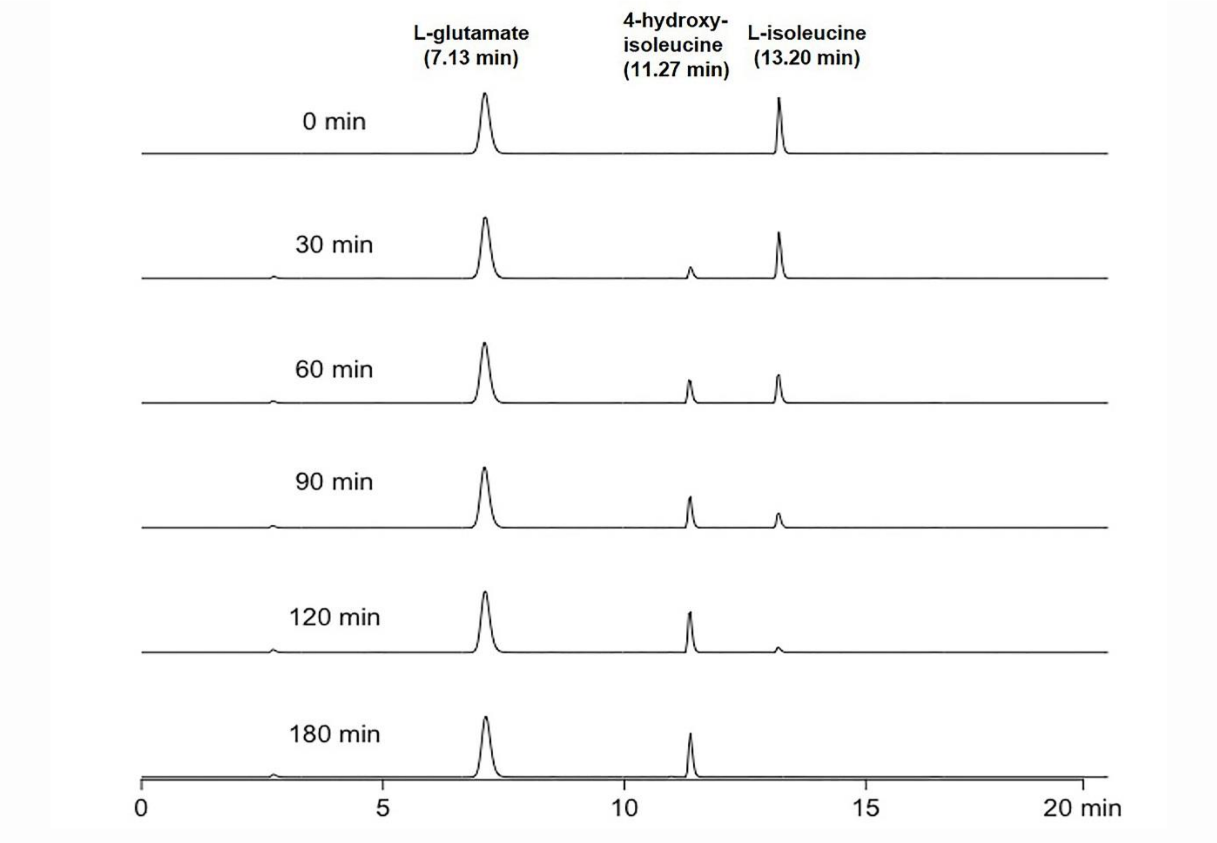

Supplementary Figure 28. HPLC analysis of the conversion from L-isoleucine to 4-hydroxyisoleucine. The reaction was performed using the L-isoleucine dioxygenase from *Bacillus thuringiensis* (BtDO) and the *C. crescentus* Weimberg enzyme cascade. After 3 h of incubation, all of the L-isoleucine (retention time 13.20 min) was converted to 4-hydroxyisoleucine (retention time 11.37 min). L-glutamate was used as reference compound with a retention time of 7.13 min. The experiment was performed without replicate (n=1). Source data are provided as a Source Data file.

Supplementary References

1. May, R. A. & Stevenson. K.J. Software review of Origin 8. *J. Am. Chem. Soc.* **131**, 872-872 (2009).
2. Archer, R. M. et al. Syntheses of 2-keto-3-deoxy-D-xylonate and 2-keto-3-deoxy-L-arabinonate as stereochemical probes for demonstrating the metabolic promiscuity of *Sulfolobus solfataricus* towards D-xylose and L-arabinose. *Chem. Eur. J.* **19,** 2895-2902 (2013).
3. Kerber, R. C. & Fernando, M. S. α-oxocarboxylic acids. *J. Chem. Educ.* **87,** 1079-1084 (2010).
4. Copper, A. J. & Redfield, A. G. Proton magnetic resonance studies of alpha-keto acids. *J. Biol. Chem.* **250,** 527-532 (1975).
5. Kuhn, R., Weiser, D. & Fischer, H. Aminozucker-Synthesen, XIX Basen-katalysierte Umwandlungen der N-Phenyl-d-hexosaminsäurenitrile. *Liebigs Ann. Chem.* **628,** 207-239 (1959).
6. Kodera, T. et al. A novel l-isoleucine hydroxylating enzyme, l-isoleucine dioxygenase from *Bacillus thuringiensis*, produces (2S,3R,4S)-4-hydroxyisoleucine. *Biochem. Biophys. Res. Commun.* **390.3**, 506-510 (2009).
7. Enoki, J., Meisborn, J., Müller, A. C. & Kourist, R. A multi-enzymatic cascade reaction for the stereoselective production of gamma-oxyfunctionalyzed amino acids. *Front. Microbiol.* **7**, 425 (2016).
