# Supplementary material for: A combined experimental and modelling approach for the Weimberg pathway optimisation
Source: Nat Commun. 2020 Feb 27;11:1098. doi: 10.1038/s41467-020-14830-y (PMC7046635; doi:10.1038/s41467-020-14830-y)
Supplement: Supplementary file 3 — Reporting Summary [file 41467_2020_14830_MOESM3_ESM.pdf]

nature research

Corresponding author(s): B. Siebers, J.L. Snoep

Last updated by author(s): 11/01/2020

Reporting Summary

Nature Research wishes to improve the reproducibility of the work that we publish. This form provides structure for consistency and transparency in reporting. For further information on Nature Research policies, see Authors & Referees and the Editorial Policy Checklist.

Statistics

For all statistical analyses, confirm that the following items are present in the figure legend, table legend, main text, or Methods section.

n/a	Confirmed	
☐	☒	The exact sample size (*n*) for each experimental group/condition, given as a discrete number and unit of measurement
☐	☒	A statement on whether measurements were taken from distinct samples or whether the same sample was measured repeatedly
☒	☐	The statistical test(s) used AND whether they are one- or two-sided<br>*Only common tests should be described solely by name; describe more complex techniques in the Methods section.*
☒	☐	A description of all covariates tested
☒	☐	A description of any assumptions or corrections, such as tests of normality and adjustment for multiple comparisons
☐	☒	A full description of the statistical parameters including central tendency (e.g. means) or other basic estimates (e.g. regression coefficient) AND variation (e.g. standard deviation) or associated estimates of uncertainty (e.g. confidence intervals)
☒	☐	For null hypothesis testing, the test statistic (e.g. *F*, *t*, *r*) with confidence intervals, effect sizes, degrees of freedom and *P* value noted<br>*Give P values as exact values whenever suitable.*
☒	☐	For Bayesian analysis, information on the choice of priors and Markov chain Monte Carlo settings
☒	☐	For hierarchical and complex designs, identification of the appropriate level for tests and full reporting of outcomes
☒	☐	Estimates of effect sizes (e.g. Cohen's *d*, Pearson's *r*), indicating how they were calculated

Our web collection on statistics for biologists contains articles on many of the points above.

Software and code

Policy information about availability of computer code

Data collection	Standard software was used for collection of RAW data from the spectrophotometer and NMR equipment and stored in Excel files.
Data analysis	For data analysis Mathematica v12 (www.wolfram.com) was used. Standard methods for parameterization of the rate equations were used, and all Mathematica notebooks and linked experimental data files are available on the FAIRDOMHub, see below in the "Data" section.

For manuscripts utilizing custom algorithms or software that are central to the research but not yet described in published literature, software must be made available to editors/reviewers. We strongly encourage code deposition in a community repository (e.g. GitHub). See the Nature Research guidelines for submitting code & software for further information.

Data

Policy information about availability of data

All manuscripts must include a data availability statement. This statement should provide the following information, where applicable:

- Accession codes, unique identifiers, or web links for publicly available datasets
- A list of figures that have associated raw data
- A description of any restrictions on data availability

For data and model management the FAIRDOMHub was used (https://fairdomhub.org). All data are made public at (https://fairdomhub.org/investigations/284). For the figures we provide DOIs as well as SED-MLs for each experiment.These links provide access to life simulations of the models, include experimental data, to reproduce all model simulation figures in the manuscript.
All source data are also provided as Excel file.

Field-specific reporting

Please select the one below that is the best fit for your research. If you are not sure, read the appropriate sections before making your selection.

☒ Life sciences ☐ Behavioural & social sciences ☐ Ecological, evolutionary & environmental sciences

For a reference copy of the document with all sections, see nature.com/documents/nr-reporting-summary-flat.pdf

Life sciences study design

All studies must disclose on these points even when the disclosure is negative.

Sample size	The study was performed with purified enzymes, and in cell free extracts of bacterial cultures, so a sample size as related to population studies is not applicable to this study.
Data exclusions	No data was excluded form the study.
Replication	The study ran over a period of three years, and during this period several rounds of enzyme purification were performed, and independent bacterial cultures were analyzed. All the experiments were reproducible, within experimental error.
Randomization	The study was performed with purified enzymes and cell free extracts of bacterial cultures, so randomization in independent groups was not applicable to this study.
Blinding	The study was performed with purified enzymes and cell free extracts of bacterial cultures, so blinding was not relevant to this study.

Reporting for specific materials, systems and methods

We require information from authors about some types of materials, experimental systems and methods used in many studies. Here, indicate whether each material, system or method listed is relevant to your study. If you are not sure if a list item applies to your research, read the appropriate section before selecting a response.

Materials & experimental systems

n/a	Involved in the study
☒	☐ Antibodies
☒	☐ Eukaryotic cell lines
☒	☐ Palaeontology
☒	☐ Animals and other organisms
☒	☐ Human research participants
☒	☐ Clinical data

Methods

n/a	Involved in the study
☒	☐ ChIP-seq
☒	☐ Flow cytometry
☒	☐ MRI-based neuroimaging
