# Supplementary material for: A combined experimental and modelling approach for the Weimberg pathway optimisation
Source: Nat Commun. 2020 Feb 27;11:1098. doi: 10.1038/s41467-020-14830-y (PMC7046635; doi:10.1038/s41467-020-14830-y)
Supplement: Supplementary file 4 — Description of Additional Supplementary Files [file 41467_2020_14830_MOESM4_ESM.docx]

**Description of Additional Supplementary Files**

**File Name: Supplementary Data 1**

**Description:** Pulse program for NMR
